# DWI and complex brain network analysis predicts vascular cognitive impairment in spontaneous hypertensive rats undergoing executive function tests

**DOI:** 10.3389/fnagi.2014.00167

**Published:** 2014-07-23

**Authors:** Xavier López-Gil, Iván Amat-Roldan, Raúl Tudela, Anna Castañé, Alberto Prats-Galino, Anna M. Planas, Tracy D. Farr, Guadalupe Soria

**Affiliations:** ^1^Experimental 7T MRI Unit, IDIBAPS, Institut d'Investigacions Biomèdiques August Pi i SunyerBarcelona, Spain; ^2^Expert Ymaging S.L.Barcelona, Spain; ^3^Group of Biomedical Imaging of the University of Barcelona, CIBER de Bioingenieria, Biomateriales y NanomedicinaBarcelona, Spain; ^4^Department of Neurochemistry and Neuropharmacology, Institut d'Investigacions Biomèdiques de Barcelona (IIBB-CSIC)Barcelona, Spain; ^5^Centro de Investigación Biomédica en Red de Salud Mental (CIBERSAM), ISCIIIMadrid, Spain; ^6^Human Anatomy and Embryology Unit, Laboratory of Surgical NeuroAnatomy, Facultat de Medicina, Universitat de BarcelonaBarcelona, Spain; ^7^Department of Brain Ischemia and Neurodegeneration, Institut d'Investigacions Biomèdiques de Barcelona (IIBB-CSIC)Barcelona, Spain; ^8^Department of Experimental Neurology, Center for Stroke Research BerlinCharité, Berlin, Germany

**Keywords:** DWI, DTI, connectomics, executive function, vascular cognitive impairment, animal models, *in-vivo* MRI, hypertension

## Abstract

The identification of biomarkers of vascular cognitive impairment is urgent for its early diagnosis. The aim of this study was to detect and monitor changes in brain structure and connectivity, and to correlate them with the decline in executive function. We examined the feasibility of early diagnostic magnetic resonance imaging (MRI) to predict cognitive impairment before onset in an animal model of chronic hypertension: Spontaneously Hypertensive Rats. Cognitive performance was tested in an operant conditioning paradigm that evaluated learning, memory, and behavioral flexibility skills. Behavioral tests were coupled with longitudinal diffusion weighted imaging acquired with 126 diffusion gradient directions and 0.3 mm^3^ isometric resolution at 10, 14, 18, 22, 26, and 40 weeks after birth. Diffusion weighted imaging was analyzed in two different ways, by regional characterization of diffusion tensor imaging (DTI) indices, and by assessing changes in structural brain network organization based on Q-Ball tractography. Already at the first evaluated times, DTI scalar maps revealed significant differences in many regions, suggesting loss of integrity in white and gray matter of spontaneously hypertensive rats when compared to normotensive control rats. In addition, graph theory analysis of the structural brain network demonstrated a significant decrease of hierarchical modularity, global and local efficacy, with predictive value as shown by regional three-fold cross validation study. Moreover, these decreases were significantly correlated with the behavioral performance deficits observed at subsequent time points, suggesting that the diffusion weighted imaging and connectivity studies can unravel neuroimaging alterations even overt signs of cognitive impairment become apparent.

## Introduction

Vascular dementia is, after Alzheimer's disease, the second most common cause of acquired dementia and covers between 25 and 30% of total dementia cases (O'Brien et al., 2003). Affecting around 30% of the population older than 80 years, the total number of people with dementia worldwide in 2010 was estimated at 35.6 million, a quantity expected to nearly double every 20 years (WHO data). Vascular cognitive impairment (VCI) defines alterations in cognition attributable to cerebrovascular causes, ranging from subtle deficits to dementia (Iadecola, 2013). Cognitive decline is characterized by the development of executive dysfunction even before other symptoms, such as memory impairment (O'Sullivan, 2004), become apparent. One of the main limitations is that by the time that the cognitive decline is clearly manifested, it might be too late to reverse the neurodegenerative process. Thus, identification of early changes associated with later development of dementia is imperative to evaluate the efficacy of any potential pharmacological therapy.

Hypertension is a major risk factor for vascular brain diseases and VCI (Hainsworth and Markus, 2008). In fact, hypertension can predict the development of dementia in almost 60% of the subjects with executive dysfunction (attention and mental processing speed) (Oveisgharan and Hachinski, 2010). Spontaneously hypertensive rats (SHR) are a very well-characterized strain, originally derived from normotensive Wistar-Kyoto rats (WKY), they are now genetically divergent and gradually develop a stable and chronic hypertension in their first 2–4 months of life (Sabbatini et al., 2002). These animals show representative symptoms of human essential hypertension and resemble most key features of human cerebral small vessel (CSV) disease (Hainsworth and Markus, 2008). Important anatomical changes have been observed in the brain of SHRs, such as ventricular enlargement, reduction of brain tissue and decreases in brain weight and gray matter volume (Tajima et al., 1993). Furthermore, SHRs exhibit poor performance in several cognitive tests including impairments in learning and memory, increased locomotor and exploratory behavior, as well as exaggerated responses to stressful stimuli (Meneses et al., 1996; Sabbatini et al., 2002; Robertson et al., 2008). Since this experimental condition resembles, to some extent, human neurodegenerative disorders with cognitive impairment (Sabbatini et al., 2002) we hypothesize that SHRs are suitable as an experimental model of VCI.

Anatomical and microstructural magnetic resonance imaging (MRI), such as diffusion tensor imaging (DTI), alterations correlate with the neuropathology in different brain disorders. Therefore, these techniques could provide non-invasive early markers of disease (Kakeda and Korogi, 2010; Hoeft et al., 2011; Román and Pascual, 2012; McMillan et al., 2013). DTI estimates the diffusion of water molecules in each voxel and produces scalar maps, including fractional anisotropy (FA), mean diffusivity (MD), axial diffusivity (AD), and radial diffusivity (RD) maps. These parameters describe the microstructure and integrity of white and gray matter in the brain in a very specific manner (Boretius et al., 2012) that allows monitoring disease progression, studying brain development, and characterizing anatomical phenotypes in the central nervous system of humans and rodents (Zhang et al., 2012). Thus, DTI is proven to be a sensitive marker of white matter damage. White matter abnormalities are often observed as hyperintensities on T2 scans (leukoaraiosis) in the brain of CSV patients (Babikian and Ropper, 1987; Hachinski et al., 1987; O'Sullivan, 2004). White matter tract damage might lead to a disconnection syndrome, where the communication between different brain structures is impaired by the disruption of cortico-cortical and cortico-subcortical connections, ultimately leading to the loss of cognitive function. However, in patients with CSV, the results in the intelligence quotient tests did not correlate with the alterations in T2 scans, while a strong correlation was demonstrated between DTI indices and cognitive dysfunction (O'Sullivan, 2004). The question that remains to be answered is whether DTI changes can be detected at very early stages of cognitive dysfunction in patients that will later develop overt signs of CSV, VCI, or Alzheimer's disease.

Diffusion weighted imaging (DWI) connectomics analysis is based on the tractographic reconstruction of neuronal connections represents a further step toward the potential use of DTI as a biomarker (Wedeen et al., 2005; Hagmann et al., 2006). In fact, connectomics assumes that the neuroanatomical connections within the brain have “small-world” properties and can be analyzed by graph theory (Hilgetag and Kaiser, 2004; Sporns and Zwi, 2004; Iturria-Medina et al., 2008; Rubinov et al., 2009). In this analysis the brain of a subject is represented as a network with modular community structure (Bullmore and Sporns, 2009); the nodes of the network are defined as anatomical brain regions, and the edges of the network are defined as the density of fiber trajectories that can be reconstructed between two brain regions. Connectomics have shown that the modularity of the human brain is altered in several neurodegenerative disorders such as schizophrenia, Alzheimer's disease, multiple sclerosis, mild cognitive impairment (for review see Griffa et al., 2013), and also in developmental disorders like autism (Li et al., 2012) or infants with intrauterine growth restriction (Batalle et al., 2012).

Obtaining a non-invasive biomarker that allows monitoring of new therapeutic drugs in a preclinical phase, using the same tools as in the clinical phases, is important to boost research in VCI. To this end, the main purpose of the present work was to demonstrate the predictive value of DTI and analysis of structural networks (or connectomics) in an animal model of VCI. We evaluated, first, if DTI and brain network features correlated with cognitive decline in SHRs. Secondly, we investigated if brain network features can predict the behavioral impairment in a longitudinal experiment, lasting over 10 months, which coupled periodic performance of complex cognitive tests (evaluating learning, memory and behavioral plasticity skills) with MRI acquisition of a high angular resolution DWI sequence.

## Materials and methods

### Animals

Experiments were performed in adult SHR and Wistar male rats 10 weeks of age, weighing 200–230 g at the beginning of the study. Rats were housed two per cage under controlled temperature (21 ± 1°C) and humidity (55 ± 10%), with a 12-h light/12-h dark cycle (light between 8:00 AM and 8:00 PM). Food and water were available *ad libitum* during all experiments except during exposure to the behavioral paradigms. Three days before starting each behavioral testing period, rats were put on to a food-restriction schedule in order to maintain them at 95% of their initial weight. Animal work was performed following the local legislation (Decret 214/1997 of July 30th by the “Departament d'Agricultura, Ramaderia i Pesca de la Generalitat de Catalunya”) under the approval of the Ethical Committee of the University of Barcelona (CEEA), and in compliance with European legislation.

### Operant conditioned behavioral tasks

#### Apparatus

Behavioral testing took place within two operant conditioning chambers (30 × 24 × 30 cm; Med Associates, Georgia, VT) in a sound-attenuating wooden box containing a fan for ventilation and to mask extraneous noise. Each chamber was fitted with two retractable levers located on either side of a centrally positioned food magazine into which an external pellet dispenser delivered 45 mg pellets (Noyes dustless pellets; Rodent grain-based diet; BioServ, Phymep, Paris, FR). Each chamber had a light-emitting diode (LED) positioned centrally above each lever, a magazine light, and a house light. Magazine entry was detected by an infrared photocell beam located horizontally across the entrance. The apparatus was controlled by MEDPCIV software.

#### Training, discrimination and reversal testing

The operant training protocols were based on those previously described (Castañé et al., 2010; Soria et al., 2013) with minor modifications. First, animals were trained under a fixed ratio 3 schedule (3 presses on a lever to obtain 1 food pellet) separately on each lever. Each rat had one training session per day and was trained to reach a criterion of nine correct trials in two consecutive blocks of 10. Once the criterion was reached, the initial discrimination test evaluated the learning skills of both groups of animals as rats had to discriminate the active and the inactive levers based in their relative position (50% of animals were trained to the left lever and 50% to the right). Four weeks later rats were retested on discrimination, which examined the memory capacity, and once the same criterion was achieved, the reversal phase started. Thus, during this phase the position of the active lever was reversed. Trials continued until achievement of the same criteria. This process, re-discrimination and reversal, was repeated after each subsequent scan time, namely weeks 18 and 22. During this testing period a light above one of the two levers was randomly shown.

#### Set-shifting task

After 26 weeks, animals underwent a final spatial response re-discrimination step, and were trained in the set-shifting task. This task required the animal to cease following an egocentric spatial response strategy and instead use a visual-cue discrimination strategy to obtain food reward (Floresco et al., 2008). The previous random light presented over the levers during the response interval was now the conditioned stimulus, which indicated the active lever. Trials were performed in a manner identical to the initial discrimination task.

### Magnetic resonance imaging

MRI experiments were conducted on a 7.0 T BioSpec 70/30 horizontal animal scanner (Bruker BioSpin, Ettlingen, Germany), equipped with an actively shielded gradient system (400 mT/m, 12 cm inner diameter). The receiver coil was a 4-channel phased-array surface coil for the rat brain. Animals were placed in a supine position in a Plexiglas holder with a nose cone for administering anesthetic (1.5% isofluorane in a mixture of 30% O_2_ and 70% N_2_O) and were fixed using a tooth bar, ear bars and adhesive tape. Tripilot scans were used to ensure accurate positioning of the head in the isocenter of the magnet.

DWI experiments were performed using an echo planar imaging DTI sequence with *TR* = 14500 ms, *TE* = 30.85 ms, four segments, *b* = 1000, 126 diffusion directions, five B0 images, FOV = 22.23 × 22.23 × 17.92 mm^3^, matrix size = 72 × 72 × 58 pixels^3^, resulting in an isometric spatial resolution of 0.309 × 0.309 × 0.309 mm^3^ and an acquisition time of 2 h 6 min.

**Figure 1:**
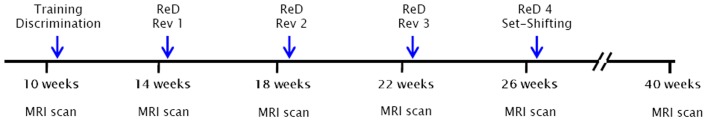
**Timeline of the experimental design**. Six MRI time points were analyzed that corresponded to 10, 14, 18, 22, 26, and 40 weeks of age. The blue arrows indicate the cognitive tests that started a week after each MRI scan. All 23 animals (11 control and 12 SHR) underwent the MRI scans. Of those same animals, 16 (8 control and 8 SHR) performed the behavioral tests. ReD, re-discrimination; Rev, reversal learning.

A scheme of the timeline for the whole experimental design is shown in Figure 1. Six MRI time points were analyzed that corresponded to 10, 14, 18, 22, 26, and 40 weeks of age. The blue arrows indicate the cognitive tests that started a week after each MRI scan. All 23 animals (11 controls and 12 SHR) underwent the MRI scans. Of those same animals, 16 (8 control and 8 SHR) performed the behavioral tests.

### Image analysis

#### Image processing, registration, and parcellation

Scalar maps, FA, MD, AD, and RD, were calculated with Paravision 5.0 software (Bruker Biospin, Etlingen, Germany) and converted to NIfTI format with custom-made programs written in Matlab (The MathWorks, Inc., Natick, MA, USA).

**Figure 2:**
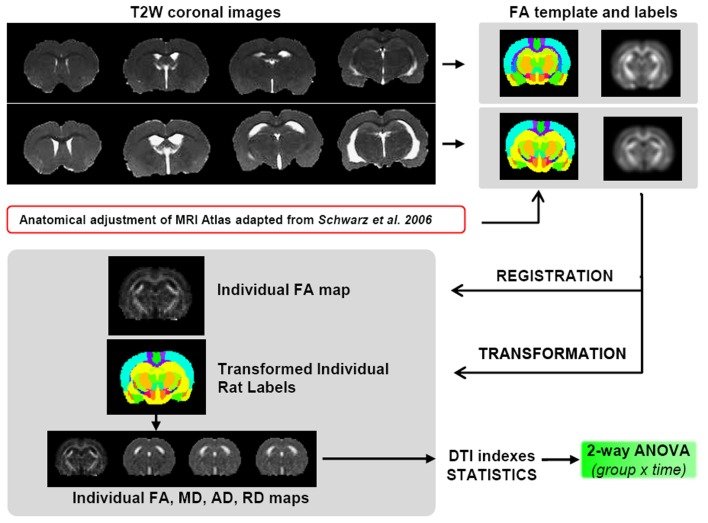
**Scheme of the DTI data processing**. SHRs have enlarged ventricles compared to control animals, thus it is not viable to perform the registration process for a unified voxel based analysis. Therefore, two different FA templates were created, one for controls and one for SHRs, including a corresponding atlas of neuroanatomical structures. To avoid interpolation of the original data, the template was registered to the individual FA maps, and this transformation was applied to the corresponding atlas to obtain the values of the 4 DTI indexes (FA, MD, AD, and RD) for both SHR and Wistar rats for each of the ROI contained in the atlas.

Due to the large difference in ventricle size between SHR and Wistar rats, all tested registration paradigms, including elastic registration, failed for a voxel-based analysis. For this reason a precise procedure was followed to obtain the best regional analysis of the DTI indexes (Figure 2). Thus, once the skull stripping was done, two separate FA average map for both SHR and Wistar rats were generated by registering and averaging the FA maps of all animals contained in each group (*n* = 12) at 10 weeks. Affine registration was used with normalized mutual information metric methods. For the definition of 3D brain structures, the rat brain atlas was modified from Schwarz et al. (2006) whose digitalized version of Paxinos and Watson (2006) had 48 composite bilateral structures. After rescaling the atlas to the current DTI resolution, we divided the original structures into left and right and performed higher level composition leaving 42 structures per hemisphere (for example, the NAcc core and shell were included in the same unit). Each brain structure was manually edited over the two FA average maps, only at this initial state, and considered a 3D volume of interest (VOI) from which DTI indices could be later obtained for each subject. The skull stripping, the registration/transformation process, the atlas edition and the extraction of the DTI scalar values were performed with specific software for scientific image processing and visualization (Amira, Visage Image, Inc.).

#### Regional analysis of DTI scalar maps

In order to avoid any type of interpolation of the original acquired data, both FA average maps were affine registered to their corresponding individual FA map at each time point and the same transformation matrix was applied to the VOIs template. In this way, all DTI indices were obtained from the original individual data for each VOI. VOIs including less than 5 voxels in the same plane were discarded since the registration did not guarantee its neuroanatomical location. Examples of the brain atlas aligned to their respective FA maps after the registration process at different time points can be seen in Supplementary Figure 1.

#### Computation of whole brain network from diffusion MRI data

Whole brain connectivity was computed according to standard procedures described in (Hagmann et al., 2007), with minor modifications:

1. *Reconstruction of water diffusion function:* water diffusion function was calculated with Q-Ball Imaging (Tuch, 2004) to resolve crossing fibers, as this is estimated to occur more than 50% of the time in the brain (Wedeen et al., 2008).
2. *Tractography:* Neural trajectories were estimated by placing multiple seeds in white matter voxels until stagnation of the process occurred. White matter voxels were estimated by thresholding Generalized Fractional Anisotropy (Tuch, 2004) within the brain mask, for each fiber orientation according to background noise levels. This threshold ranged from 0.026 to 0.029 with no significant differences observed between groups (*p* = 0.56). Minimum length of accepted trajectories was set to 3 voxels (about 0.9 μm). Trajectories were computed following an adaptive 4th-order Runge-Kutta (Basser et al., 2000).
3. *Fiber filtering:* trajectories were statistically studied using a custom-made algorithm based on a previous report (Gigandet et al., 2008) to remove those unlikely.
4. *Computation of graph model:* As brain parcellation and tractography were both computed in diffusion space, no registration was required for quantification. Weight of edges was computed as the ratio between the number of fibers connecting two brain regions (nodes) and the total white matter volume (equal to the number of positions that permitted seed placing). This pipeline allowed computing individualized adjacency matrices to represent the white-matter connectivity in whole brains.

#### Graph analysis

Subsequent graph analysis after tractography and brain parcellation offers a more robust mathematical approach and an integrated picture of the brain (instead of pin-point different regions we obtain an integrated evaluation). The present study has the limitation of low number of samples (16 samples from 3 different time points), and our connectivity matrix has 3486 values, which defines a multivariate space with high dimensionality. For this, graph analysis represents an approach to further boil down such complexity into a few values that can be statistically analyzed with certain confidence. In fact, we divided our graph analysis at two standard levels: global and regional parameters.

1. *Global parameters* included three aspects: global and local efficiency of transfer of information, and hierarchical modularity. Global efficiency is the average inverse shortest path length between all pairs of nodes in the network (Latora and Marchiori, 2001). Local efficiency is the average efficiency of local subgraphs and its expression is shown below. The definition used in this study is a generalization of clustering coefficient (Watts and Strogatz, 1998) for weighted networks (Onnela et al., 2005). In this work, we use the recently developed concept of nested modularity (Sales-Pardo et al., 2007) to estimate the quality of modularity based on the Q parameter, which was calculated using a recently developed multi-level method (Blondel et al., 2008). This method decomposes a network into a complete hierarchical subset of communities and takes advantage of the hierarchical organization of complex networks by using an iteratively multi-level optimization algorithm to maximize the modularity parameter Q (Salvador et al., 2005; Meunier et al., 2009).
  Regional parameters compute the role of a specific region in transferring of information according to its wiring with other regions.
2. *Local efficiency* is the individual efficiency of local subgraphs computed as is the unweighted (topological) clustering coefficient renormalized by the average intensity of “triangles” at the region. The mathematical expression is (Onnela et al., 2005):
  (1) $$E_{REG,i}=\frac{2}{k_{i}\left(k_{i}-1\right)}\sum_{j,l}{\left(w_{ij}w_{jl}w_{li}\right)}^{1/3}$$
  where *k*_*i*_ is the degree of node *i* and *w*_*ij*_ is the weight between regions *i* and *j*.

### Statistical analysis

#### Behavioral data and regional analysis of DTI indexes

Behavioral data (total trials to criteria) and DTI indexes were analyzed using GraphPad Prism software. Total trials to criteria performed during discrimination and reversal tests and DTI indexes in the selected structures were subjected to repeated measures ANOVA where the between-subjects factor was the rat strain (Wistar or SHR) and time as within-subjects factor of variation. For the total trials to criteria performed in the set-shifting test an unpaired Student's *t*-test was used to evaluate significant differences between SHR and Wistar rats. When significant overall interactions were found, further analyses of partial interactions were carried out. *Post-hoc* analyses for intergroup comparisons were performed with Bonferroni's test, which also corrects for multiple comparisons, when the initial *p*-value was significant. Differences were considered significant if *p* < 0.05. All results are expressed as mean ± standard error mean (s.e.m.).

#### Association of global parameters to study groups

We evaluated the relationship of global parameters (hierarchical modularity, global efficiency and regional efficiency) with experimental groups. A two-sided non-parametric test (Mann-Whitney U) was used to evaluate significance between groups at different time points (weeks). We also evaluated the longitudinal changes of global parameters within groups to check whether any of the parameters was statistically preserved over time. Multivariable analysis was carried out with MANOVA to evaluate discriminant ability of the global parameters in Matlab (The MathWorks, Inc., Natick, MA, USA).

#### Predictive model of behavioral outcomes based on regional network parameters

As global parameters showed a large association with the study groups, we evaluated whether regional network parameters were able to predict outcomes of the neurobehavioral tests. We defined 2 different statistical settings and computed different models by gathering data on a week or integral approach, which provided 3 sets of 16 animals (weekly model) and 1 set of 48 animals (integral). Due to data limitations, week models were tested in a four-fold cross validation (CV) scheme, meaning 12 samples for training and 4 for testing, and integral models were tested in a three-fold CV scheme, meaning 32 samples for training and 16 for testing.

Basic cross validation was iterated 1000 times to estimate the statistical behavior of the model and was equally employed in both statistical settings. The procedure is summarized as follows: (1) a random subset for training the model was obtained from the whole set (week/integral); (2) dimension reduction was computed by keeping the 10 principal components of training data; (3) discriminant analysis on study groups provided the most discriminant projection axis of training data; (4) we kept the 16 regions that contained the most energy on the most discriminant axis; (5) we computed a general linear model based on the 16 regions on the results of the neurobehavioral tests on the training data; (6) the obtained model was then applied to the test data and results were stored; (7) after 1000 iterations all tested data out-of-the-bag was compared to the ground truth by Pearson's correlation.

## Results

### Learning, memory, and executive function evaluation

**Figure 3:**
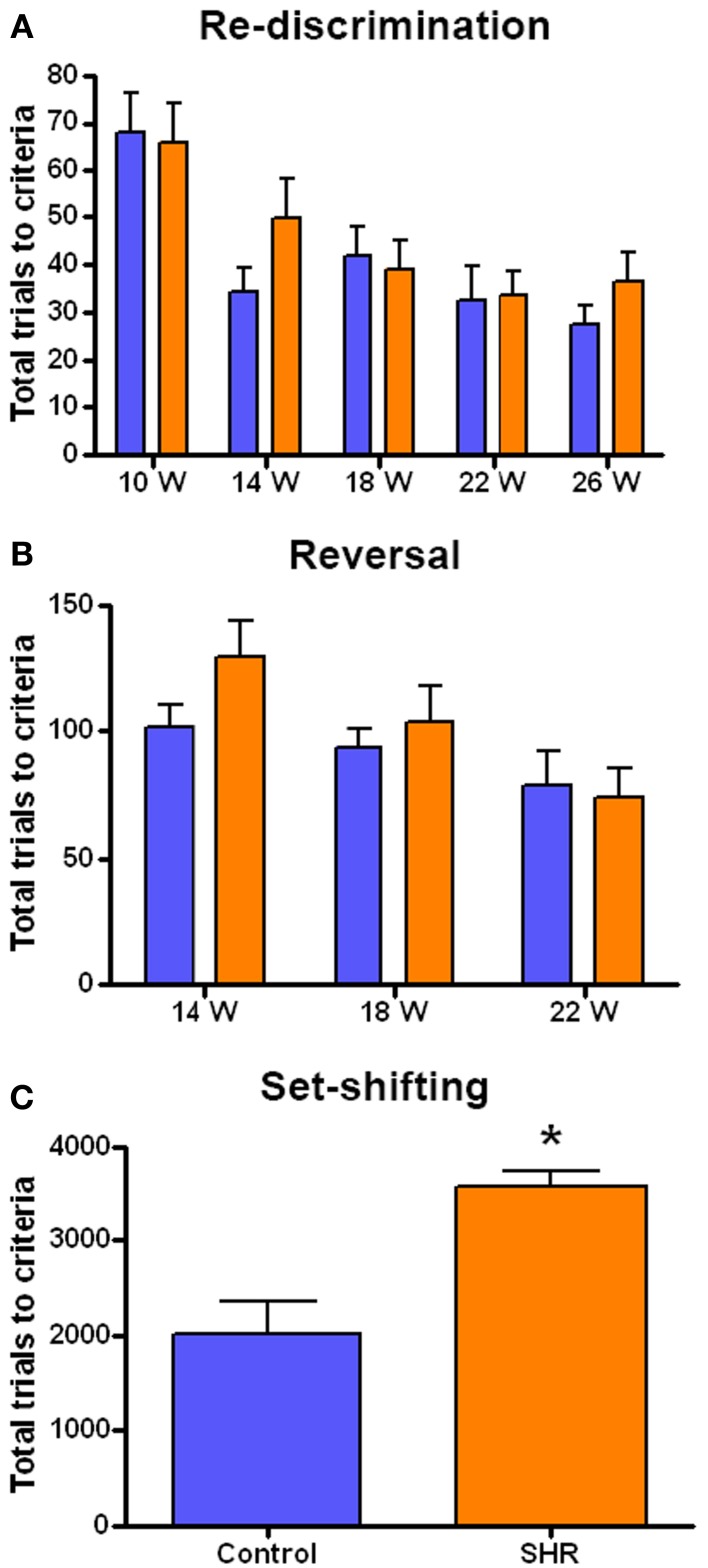
**Number of trials to reach criteria in the behavioral tests. (A)** Discrimination (10 weeks) and re-discrimination (14, 18, 22, and 26 weeks); **(B)** reversal learning, and **(C)** set-shifting test. Blue bars represents control animals (*n* = 8) and orange bars SHR (*n* = 8). Data is represented as mean ± s.e.m. Asterisks indicate differences between groups (Bonferroni test). ^*^P < 0.01.

The performance in each cognitive task is represented as the number of trials needed by the SHR (*n* = 8) and control (*n* = 8) rats to reach the criteria. We observed a similar performance in the discrimination and re-discrimination tasks (Figure 3A) as Two-Way ANOVA showed no group effect [*F*_(1, 70)_ = 0.9749; n.s.]. However, we found a significant effect of time [*F*_(4, 70)_ = 9.265; *P* < 0.001], though there was no interaction [*F*_(4, 70)_ = 0.6977; n.s.]. These results revealed no differences in learning and memory skills between the two groups and showed a time effect of learning in both SHR and Wistar rats since fewer trials to re-discriminate were needed over time.

As the rats reached each re-discrimination criteria, reversal learning was performed at weeks 14, 18, and 22 (Figure 3B). This test evaluated the behavioral flexibility as the position of the correct lever was changed. Although a significant effect of time was observed [*F*_(2, 42)_ = 5.218; *P* < 0.01] there was no significant effect of group [*F*_(1, 42)_ = 1.216; n.s.] nor interaction [*F*_(2, 42)_ = 0.9092; n.s.]. This again indicates that progressively fewer trials were required for the animals to learn the reversal.

After the fourth re-discrimination, performed at 26 weeks, the set-shifting test started (Figure 3C). This is a more difficult task in which the rats had to learn that the randomly presented light in the previous phases was now the key stimulus. Thus, this test required the animal to shift from a strategy based on the relative position to visual discrimination. Interestingly, in this case the SHR group needed significantly more trials to acquire the criteria compared to control animals, as revealed by the t-student analysis, [*T*_(7, 14)_ = 4.052; *P* < 0.01].

### Regional analysis of DTI scalar maps

**Figure 4:**
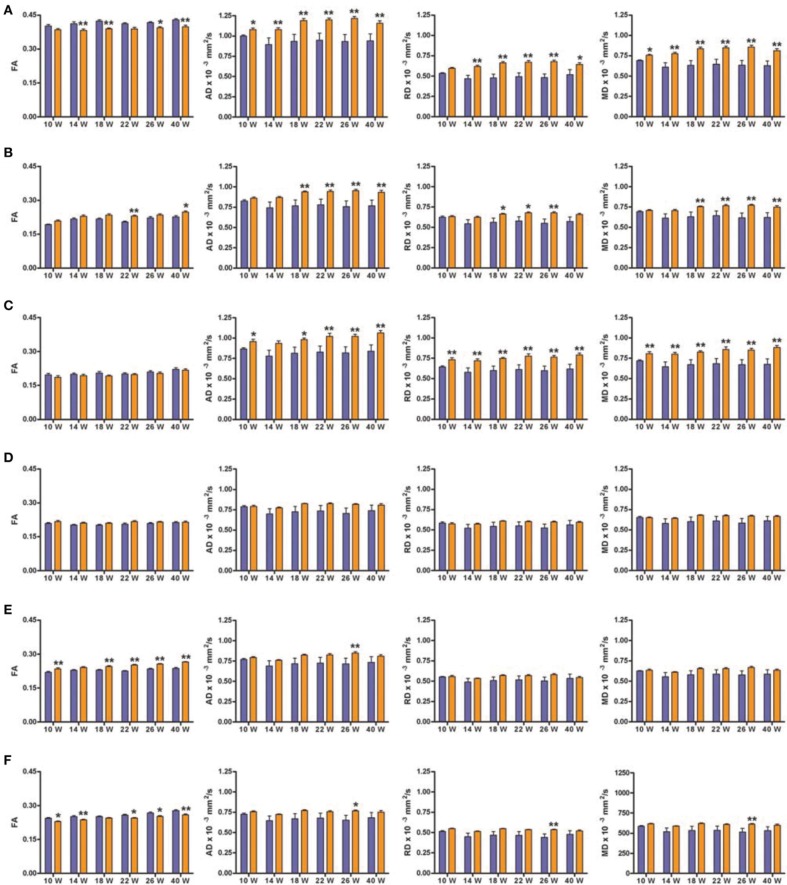
**DTI scalar map analysis**. The four indices FA, AD, RD, and MD are indicated for each of the six structures analyzed at each of the six scanning time points. Blue bars represents control animals (*n* = 11) and orange bars SHR group (*n* = 12). **(A)** Corpus Callosum, **(B)** medial Prefrontal Cortex, **(C)** Hippocampus antero-dorsal, **(D)** Orbitofrontal Cortex, **(E)** Striatum, **(F)** Nucleus Accumbens. Data is represented as mean ± s.e.m. Asterisks indicate differences between groups (Bonferroni test) ^*^P < 0.05, ^**^P < 0.01.

We will only describe six of the possible 42 structures as they are known to be related to the cognitive function evaluated in the behavioral tests: corpus callosum (CC), medial prefrontal cortex (mPFC), anterodorsal hippocampus (adHpc), orbitofrontal cortex (OFC), the striatum and the nucleus accumbens (NAcc). Figure 4 shows the four DTI indexes of each structure for the right hemisphere of SHR (*n* = 12) and control (*n* = 11) rats at all-time points measured. Supplementary Table 1 summarizes the Two-Way ANOVA results for each structure.

Significant decreases in the FA values of the CC were observed in SHRs compared to control animals at most time points (Figure 4A), with significant effects of group and time, but no interaction, suggesting a similar evolution of both groups over time. In addition, AD and MD were significantly increased in the CC of the SHR group compared to the control group at all studied time points. A significant increase of RD in the SHR group was observed from week 14, with a significant effect of group, suggesting that the RD increase did not depend on time.

FA significantly increased in SHRs compared to control rats at 22 and 40 weeks, in the mPFC (Figure 4B), a high-level association brain area related to stimulus-reinforcement learning. There was a significant effect of group and time but no interaction. The same results were obtained for the RD, where *post-hoc* analysis revealed significant increases in the SHR group at 18, 22, 26 weeks compared to controls. Similarly, significant increases in AD and MD were observed from 18 weeks in SHRs. In this case, there were significant effects of group, time and the interaction between the two factors, suggesting a group effect dependent on time.

The hippocampus, a key structure for memory-related processes, showed similar FA values in the anterodorsal portion during the whole study (Figure 4C). However, AD, RD, and MD were significantly increased in SHRs compared to control rats at almost all time points. This increase in diffusivities was not dependent on time.

In the OFC (Figure 4D), which is involved in decision-making processes, FA showed a significant effect of group, with no effect of time or interaction between the groups. In the case of RD and MD, there was a significant effect of time while AD showed significant effects of group and time but no interaction. *Post-hoc* analysis did not reveal any difference between control and SHR groups at any time point in any of the DTI indexes, suggesting that subtle differences at the various time points could account for the global effects previously described.

The striatum receives massive projections from the cerebral cortex and one of its main roles is to equilibrate motor behavior drifted by motivation. FA was significantly increased in the SHRs compared to control rats at most time points in the striatum (Figure 4E), with significant effects of group and time but no interaction between the two factors. Similar results were found for AD and MD. However, *post-hoc* analysis of AD showed a significant increase in the SHR compared to control animals only at 26 weeks, while no significant differences were obtained between control and SHRs at any time point for MD values. RD showed a significant effect of time but no effect of group or the interaction.

The NAcc, functionally related to reward and learning process, showed a significant increase of FA values in SHR compared to controls at most time points (Figure 4F). Thus, there was a significant effect of group and time but no interaction. In addition, significant increases in AD, RD, and MD were observed in the SHRs at 26 weeks of age compared to control animals. There were significant main effects of group for the three indexes, an effect of time for MD, while no interactions were observed.

### Global analysis of network-based structural connectomics

**Figure 5:**
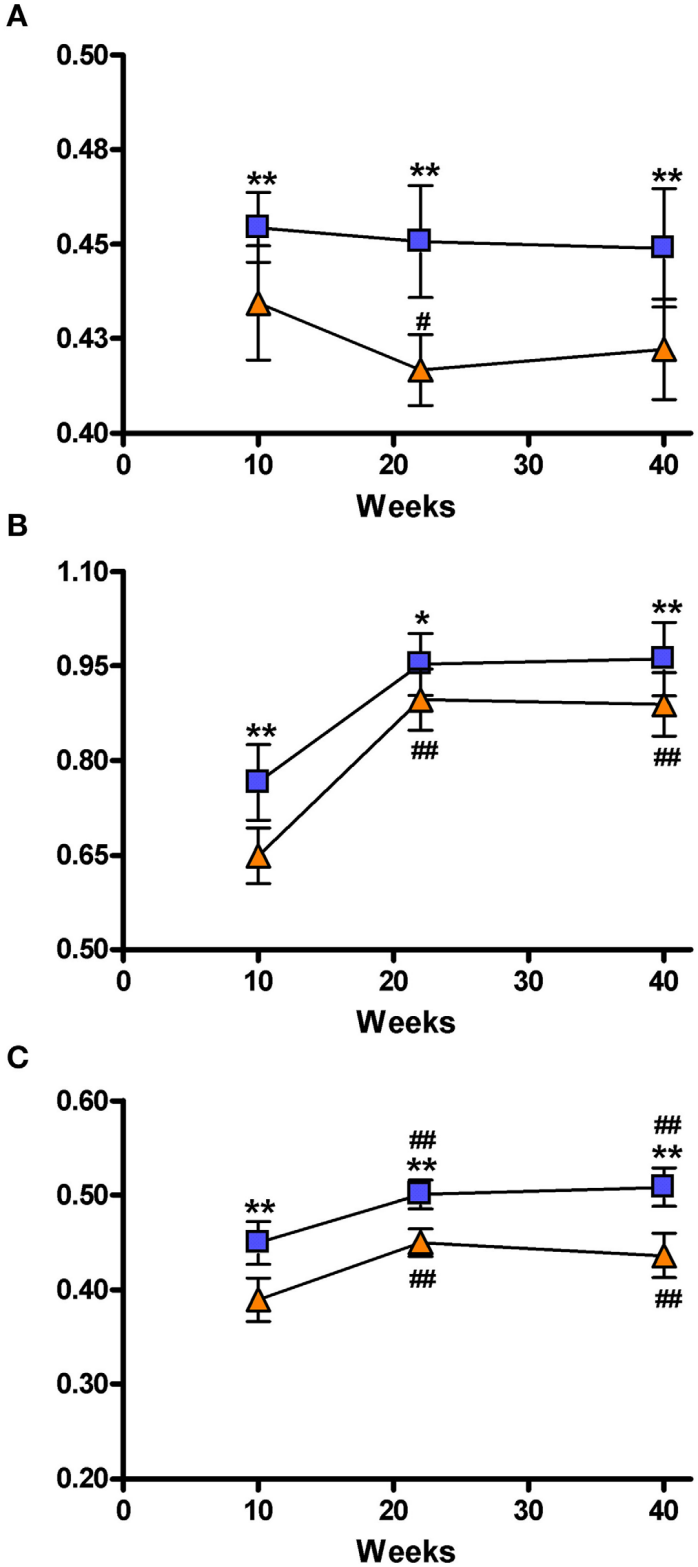
**Representation of the brain network connectivity**. Network connectivity indices at 10, 22, and 40 weeks. **(A)** Hierarchical modularity; **(B)** Global Efficiency; **(C)** Local efficiency. The blue squares represents the control animals and the orange triangles the SHRs. Data is represented as mean ± s.e.m. Asterisks indicate differences between groups (Bonferroni test) ^*^P < 0.05, ^**^P < 0.01. Hash tags indicate differences to 10 weeks (Bonferroni test) ^#^*P* < 0.05, ^##^*P* < 0.01.

Figure 5 shows the three parameters describing the global characteristics of the brain network that were obtained from graph analysis: hierarchical modularity, global and local efficiency. Because of the elevated complexity of this analysis, 3 out of the 6 acquired time points were studied, namely: 10, 22, and 40 weeks of age. Hierarchical modularity was significantly lower in the SHRs compared to control rats at all three time points. The Two-Way ANOVA revealed a significant effect of time [*F*_(2, 28)_ = 39.550; *P* < 0.001] and group [*F*_(1, 28)_ = 3.462; *P* < 0.05], although no interaction was obtained [*F*_(2, 28)_ = 1.308; n.s.]. *Post-hoc* analysis did not show significant differences over time in Wistar rats, though the SHRs showed a significant decrease of hierarchical modularity at 20 weeks (*P* < 0.05), and a clear tendency at 40 weeks compared to 10 weeks (Figure 5A). Moreover, a color-coded matrix of individual hierarchical modularity (10 weeks) was constructed in order provide an overview of the homogeneity between groups (Supplementary Figure 2). This shows that controls have an average similarity of 0.61 with a standard deviation of 0.028, whereas SHRs show an average similarity of 0.54 with standard deviation of 0.042, which is 50% higher, suggesting certain heterogeneity within the SHR group that should be confirmed with further experiments using larger data set.

Global efficacy was also lower in SHRs compared to control rats at all 3 time points (Figure 5B). There was a significant effect of time [*F*_(2, 28)_ = 151.7; *P* < 0.001] and group [*F*_(1, 28_ = 16.60; *P* < 0.001] but no interaction [*F*_(2, 28)_ = 2.375; n.s.]. *Post-hoc* analysis revealed a significant increase of global efficacy at 20 and 40 weeks compared to 10 weeks (*P* < 0.001) of age in both control and SHR groups.

Similarly, SHRs showed significantly lower local efficacies compared to control animals at the 3 analyzed time points (Figure 5C). There was a significant effect of time [*F*_(2, 28)_ = 71.26; *P* < 0.001] and group [*F*_(1, 28)_ = 58.54; *P* < 0.001], but no interaction between both factors [*F*_(2, 28)_ = 2.050; n.s.]. Again, *post-hoc* analysis showed a significant increase of local efficacy at 20 and 40 weeks compared to 10 weeks (*P* < 0.001) in both control and SHR groups. Supplementary Figure 3 shows these local efficiency differences by color coding the different VOIs in a set of coronal slices.

**Figure 6:**
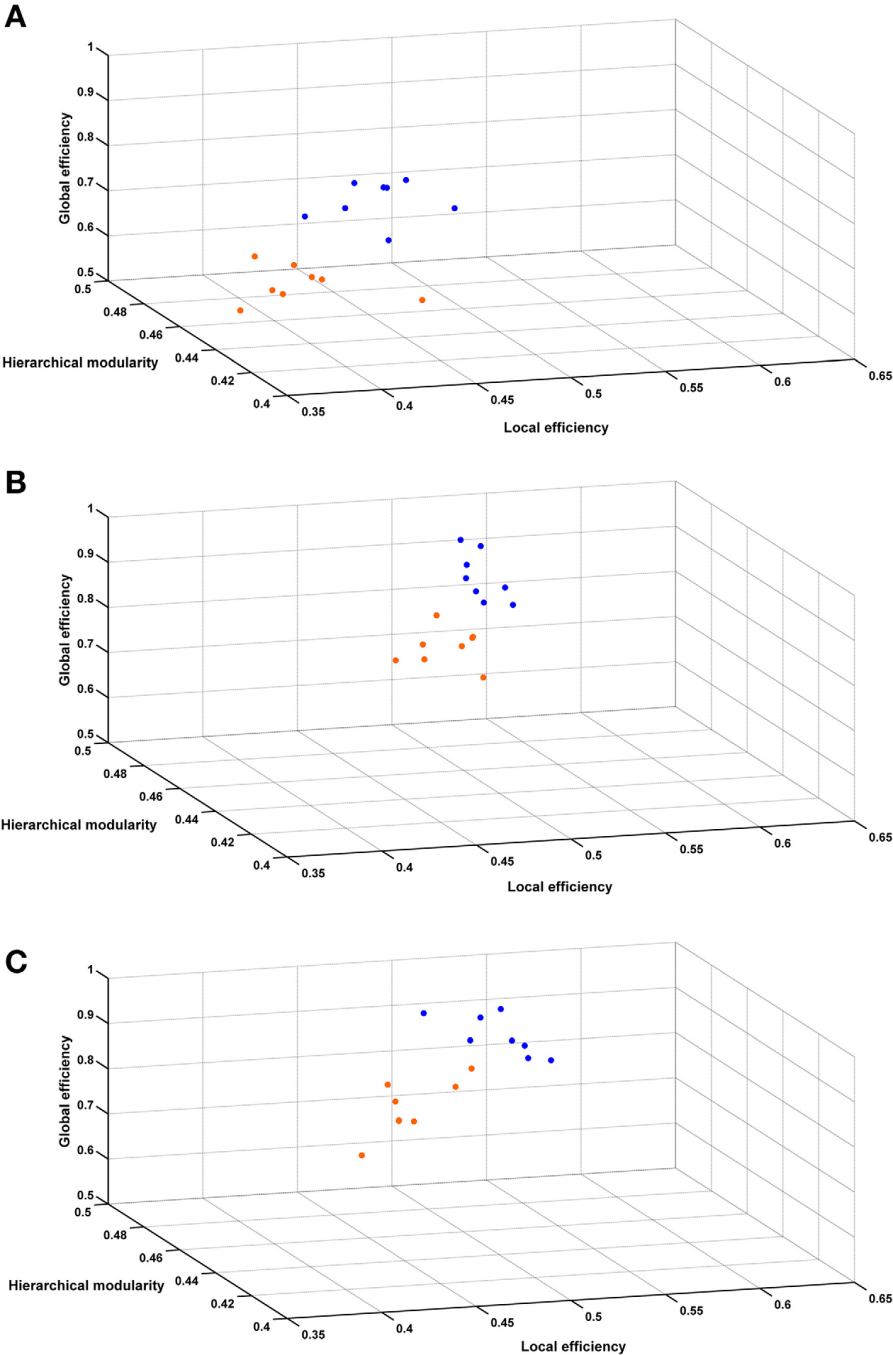
**Hierarchical modularity, global and local efficiency indices**. Three-dimensional scatter plots of all animals represented by their connectivity indexes at **(A)** 10 weeks, **(B)** 22 weeks, **(C)** 40 weeks. Blue dots represent control animals and orange dots represent SHRs.

**Figure 7:**
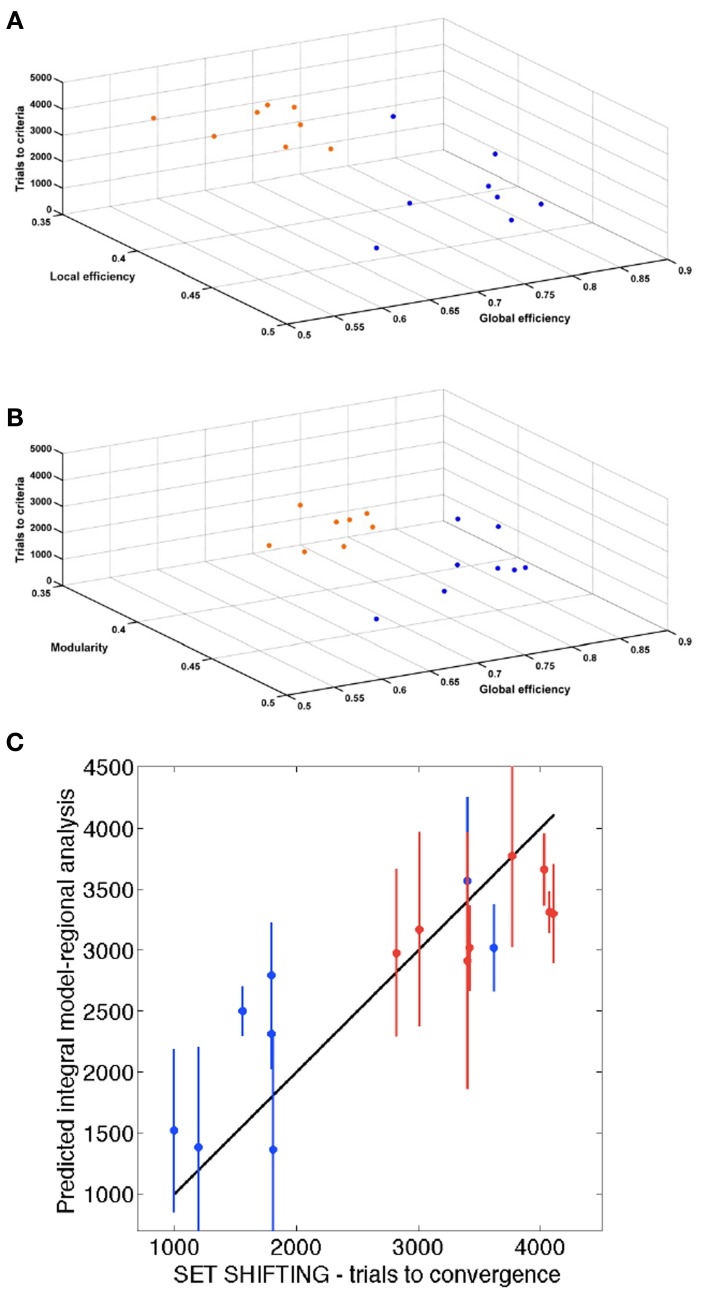
**Association of behavior and connectivity indices. (A,B)** Scatter plots representing all the animals with the number of trials required to reach criteria in the set-shifting test on the Y axis, and the connectivity indexes in the X and Z axis. Blue dots represent control animals and orange dots represent SHR. **(C)** Estimation of the final behavioral flexibility score of each animal based on the connectivity indexes at 10 weeks.

Figure 6 shows the 3 global network-based measurements in a 3D plot for each analyzed time point. A significant (*p* < 0.01) difference was found between SHR and control groups allowing a very good discrimination between these groups. In Figure 7, the behavioral data at 40 weeks (trials to criteria from set-shifting) is presented as function of the 3 global network based measurements from week 10. The results of this analysis show a greater separation between the two populations, and more interestingly, indicate the potential use of network analysis as an early predictor for the hypertension-induced behavioral deficits.

### Regional analysis of network-based structural connectomics

To assess the relationship between the results of the functional tests and those of regional analysis, we evaluated the predictive ability of a mathematical model based on the network parameters and the set-shifting outcome. We performed a three-fold cross-validation analysis to estimate the number of trials required to reach criteria in the set-shifting test (Figures 7A,B). A significant association was found (*P* < 0.001) with a Pearson's correlation of 0.89. Figure 7C shows the correlation between trials to criteria performed in the set-shifting test and the same information predicted by the integral regional model.

The feature selection block of the model learning selected 3 regions of the right hemisphere: entorhinal cortex, caudate-putamen and parietal association cortex with a frequency of 83.7, 62.7, and 57.4%, respectively. Supplementary Figure 4 shows in a representative rat of each grup the tracts emerging from mPFC and the tracts between enthorinal cortex and adHpc, one of its main target projections.

## Discussion

We studied, in a well-known animal model of chronic hypertension, the SHR, brain structural changes related to cognitive dysfunction. In this work we present longitudinal DTI and global network analysis results that are correlated with behavioral data from cognitive flexibility paradigms. Indeed, structural changes were evident weeks before the cognitive decline was apparent, and more interestingly, both DTI and brain networks studied by structural connectomics were found to be predictors of executive dysfunction.

SHRs have been previously suggested to be an experimental model of vascular dementia or VCI (Sabbatini et al., 2002; Amenta et al., 2003). Our experiment confirms their utility. There was no impairment in the SHRs' initial learning ability, as performance was similar to that of controls during the first discrimination test (Figure 3A, 10w). Subsequently, SHRs showed a slight tendency to need more trials to achieve the first re-discrimination (Figure 3A, 14w) and the first reversal (Figure 3B, 14w) compared to control animals. However, a significant group effect was only observed in the number of trials to reach criteria of the set-shifting test. Although both paradigms evaluate executive function, the set-shifting test demands a higher level of behavioral flexibility and the SHRs fall short in that respect. In their juvenile age, SHRs have been used as a model of attention deficit and hyperactivity disorder (ADHD) (for review, Sagvolden et al., 2005). Thus, although we have demonstrated similar learning and memory abilities in SHR and Wistar rats, we cannot exclude that neurocognitive deficits, secondary to those induced by hypertension, lead to the impaired behavioral flexibility observed in the set-shifting test. Other authors have reported decreased learning and memory abilities in SHRs from 2 to 18 months of age (Meneses et al., 1996) and an additional increase of perseverative and regressive errors in reversal learning and attentional set-shifting in 5- (Cao et al., 2012) and 9-week-old animals (Kantak et al., 2008). These discrepancies might be explained by the sensitivity of the different behavioral tests and by the use of Wistar rats as controls in the present study while WKY are typically the normotensive-control group for SHR. However, we, and others, observed a reduced level of locomotor activity and investigatory behavior in WKY rats compared to Wistar rats (Hård et al., 1985; Paré, 2000; Li and Huang, 2006; Cheng and Li, 2013), which makes them poor behavioral controls when compared to Wistars (Hernandez et al., 2003; Kantak et al., 2008; Cheng and Li, 2013). Arguably, there is no perfect control for the SHR in terms of behavior and with respect to the brain atrophy that is selectively observed in this strain. SHR breeding has led them to become genetically divergent even from the WKY rats. This lack of appropriate control remains a limitation when studying SHRs. Studies in SHRs comparing the effects of chronic anti-hypertensive therapy might help sorting this problem out.

The study of the executive function is especially relevant, as human VCI is characterized by the development of executive dysfunction before other symptoms such as memory impairment (O'Sullivan, 2004). These cognitive processes are dependent on the integrity of specific brain areas that we further studied by regional analysis of DTI images. We observed a consistent increase of both AD and RD in the CC, mPFC and adHpc. These brain regions are part of the neurobiological substrate underlying the executive function (Floresco et al., 2008; Castañé et al., 2010). Notably, significant changes between SHR and control rats were observed already at 10 weeks of age in the CC and adHpc, while in mPFC (a high-level association area) the increase in AD and RD was observed after 18 weeks of age. This finding suggests that a certain degree of structural damage is already present in the SHRs before the behavioral impairment is detected and we cannot exclude the possibility that this is due to their divergent genetic background when compared to the Wistar rats. We also observed interesting early FA changes in the NAcc and striatum, which is not surprising given their importance in complex behavioral tasks that require cognitive flexibility and changes in strategy, as previously reported (Floresco et al., 2006; Ragozzino, 2007; Castañé et al., 2010; Lindgren et al., 2013). In the present study, FA was more sensitive than AD and RD, which showed very little change, in revealing microstructural alterations induced by hypertension. This finding might be explained by the very rigorous DWI acquisition design followed in our study, which includes a non-interpolated isometric resolution and the use of 126 gradient diffusion directions.

However, our regional analysis of the DTI scalar maps was not devoid of some limitations. As mentioned previously, the enlargement of the ventricles prevented, even with elastic algorithms, having good registration results and performing a voxel-based analysis. Therefore, there were some areas excluded from the statistical analysis. Some recent publications showed successful image registration between brains of different mice strains by using diffeomorphic algorithms (Dodero et al., 2013; Tucci et al., 2014). While this was carried out for volumetric analysis of the structures, regions of interest were manually drawn in the DTI parametric maps such as FA (Dodero et al., 2013). Also, a separate average template for Bfc/+ and control mice was used in the case of Tucci et al. (2014), a similar protocol to the one we have used here, supporting the current procedure. Nevertheless, future studies would benefit from diffeomorphic algorithms. Another limitation was the error introduced in the measurements by the partial volume effect. The rat brain atlas was modified from Schwarz et al. (2006) whose spatial resolution was 0.19 × 0.19 × 0.8 mm^3^, while our reference FA images were 0.3 mm^3^. Since rescaling of the two resolutions was insufficient to obtain a satisfying result, a dedicated edition of the atlas was prepared in order to adjust the VOIs to the present resolution. Therefore, although affecting similarly both SHR and Wistar rats, partial volume effects may be most prevalent in smaller structures. Indeed, all these limitations might explain the fact that some statistical differences were lost at the last time point since the size of the brain had considerably increased compared to the initial scans. Nevertheless, our results agree with other studies showing reduced volume of striosomes, frontal and occipital cortex and hippocampal gray and white matter (Sabbatini et al., 1999, 2001; Amenta et al., 2003). In summary, DTI scalar maps obtained longitudinally in SHRs showed alterations in white and gray matter structures at early stages of the VCI phenotype in SHRs. Moreover, these results suggest the potential predictive value of these measurements in an animal model of VCI, which might be a crucial step for the development and monitoring the effects of potential pharmacologic treatments.

Executive function includes several neuronal systems working in coordination. Thus, we also wanted to study the emerging behavioral phenotype from a global network perspective. Potent computational techniques to study brain networks and connectivity have been developed recently to investigate the so called human “connectome” (Sporns, 2011). In general terms, the brain of mammals show small-world topology, which is represented by equilibrium between global and local information processing, sparse connectivity between nodes and low wiring costs (Bassett and Bullmore, 2006; Iturria-Medina et al., 2008). In this line, hierarchical modularity provides information of the degree to which a network may be subdivided into modules or communities of the brain's functional organization, where basic structural subnetwork modules correspond to specific functional domains (Chen et al., 2008; Iturria-Medina, 2013). The global efficiency reflects the potential for parallel exchange of neural information between involved anatomical regions, while the local efficiency reflects the potential tendency for communities to exist or clusters of different regions that deal with common neural information. This entire new paradigm for studying brain connectivity is contributing to the understanding of pathophysiological mechanisms, and supporting in general the hypothesis that network randomization and subsequent loss of optimal organization could be a common final result of the brain's reaction to lesions or neurodegenerative processes (Wang et al., 2010; Iturria-Medina, 2013). Indeed, this is the approach we have also used in our work to study hypertension effects on brain connectivity and their correlation with cognitive decline in behavioral flexibility. To our knowledge this is the first DWI connectomics study performed in an *in vivo* rat experimental disease model. We have shown that hierarchical modularity was lower in SHRs compared to Wistar rats at all measured time points. This finding is aligned with our expected hypothesis as modularity is a well-known feature of complex systems, moreover, the higher the modularity the higher the adaptability of the system. Furthermore, hierarchical modularity is a fairly constant feature throughout longitudinal analysis and it is the only value that did not significantly change within the same group over time, but it was significantly different between SHR and Wistar rats. Global and local efficiencies are highly related to “density” of neural connections and are expected to grow during development and achieve a plateau after that. In this line, a recent study reported developmental changes assessed by MRI in rats occurring up to 12 weeks after birth (Mengler et al., 2013). This might explain the progression of both efficiencies observed in both SHR and Wistar rats between 10 and 22 weeks (Figures 5B,C). Another hypothesis to explain this evolution might be that brain plasticity developed through repetitive behavioral testing. Several studies have demonstrated microstructural changes in white and gray matter by DTI after short and long-term behavioral training (Scholz et al., 2009; Takeuchi et al., 2010; Sagi et al., 2012; Hofstetter et al., 2013). Moreover, both local and global efficiency were found to be significantly lower in SHRs compared to controls, which was also expected as a higher global efficiency should be present in healthy subjects and is associated with a more efficient transmission of the information within two random regions of the brain. A higher local efficiency is related to higher resiliency, since a more robust communication between regions of the same community suggests that the brain wiring is configured in order to handle disruptions or insults in a better manner. Therefore, our results suggest that SHRs have a brain network in which connectivity between brain regions follows a more random pattern. In a compensatory manner, the brain would increase the number of connections to “bridge” those regions affected by the disease, but in a less organized manner. This would converge into an overall lower hierarchical modularity. Interestingly, by representing the three global network measurements in the same scatter diagram, a clear separation of the two strain populations was observed at 10, 22, and 40 weeks (Figure 6). Therefore, we evaluated the accuracy of such global network parameters to be used as predictors in 2 different manners, individually and as a composite measure. These results were compared to the DTI scalar maps at 10 weeks. Receiver operating characteristics (ROC) curves and accuracy data can be found in Figure 5 and Table 2 of Supplementary material. Global network parameters showed the highest accuracy discriminating both groups of rats (area under the ROC curves in the range of 0.81–0.94% for the global network measures, compared to 0.21–0.90 for the DTI scalar maps). Moreover, a stronger spatial separation between SHRs and Wistar rats was observed if the number of trials to criteria performed in the set-shifting tests was represented as a function of hierarchical modularity, global and/or local efficiency at 10 weeks of age (Figure 7). These findings support the hypothesis that DWI connectomics and subsequent global network analyses are an appropriate non-invasive imaging biomarker of VCI.

Certainly, behavioral flexibility, an example of executive function, relies on an effective brain circuit composed of healthy brain structures. In our imaging protocol, the tractography calculated for the whole brain was parceled into 42 functional neuroanatomical structures per hemisphere. We used this information to perform the regional analysis of network-based structural connectomics at 10 weeks, used for the estimation of the final behavioral flexibility score of each animal (Figure 7C). Our model predicted very precisely the eventual behavioral score with a Pearson's correlation of 0.89. Therefore, not only is DWI connectomics able to discriminate between pathological cases and controls, but it is also informative of the future behavioral function.

Notwithstanding, some limitations need to be discussed at this point. We cannot exclude that the reduced structural connectivity observed in SHRs may be secondary to gray matter volume differences/atrophy. Indeed, in agreement with previous studies (Tajima et al., 1993), ventricle volumes (Supplementary Table 3) in SHRs were bigger compared to Wistar rats. However, brain atrophy in SHRs was only found after 3 months of age (Bendel and Eilam, 1992; Tajima et al., 1993). In this line and in view of our findings on structural connectivity, the alterations seem to be more complex than just gray matter loss. Further investigations need to be carried out to elucidate if brain atrophy is contributing, and to what degree, to the connectivity differences we have reported. For instance, tract based spatial statistics; a voxel-based analysis of diffusion data (Smith et al., 2006), could complement the integrative point of view of the current graph theory analysis by adding regional information to the differences in white matter tracts.

In conclusion, our study demonstrated first, that SHRs is a good model for the investigation of VCI on account of the evolution of cognitive decline (executive function) as well as changes in the brain microstructure and connectivity. Second, DTI scalar maps and network-based structural connectomics reveal neuronal alterations before the onset of the executive function impairment. Third, the accuracy of network-based structural connectomics for discrimination between SHRs and Wistar rats is higher than DTI scalar maps. Finally, DWI structural connectomics not only significantly correlated with the cognitive behavioral deficit, but was able to predict the level of future impairment in our experimental model. Further experiments are needed to complement and validate these findings. However, our results strongly support the use of structural brain connectomics in the preclinical investigation for the development and treatment of neurodegenerative diseases, which might lead to an earlier clinical diagnosis and increase the effectiveness of any potential treatment.

## Author contributions

Xavier López-Gil carried out the MRI acquisitions, the DTI analysis, the behavioral studies, analyzed the data and wrote the paper. Iván Amat-Roldan carried out the structural based connectomics analysis. Raúl Tudela contributed with custom-made programs for the analysis of DTI and help with the interpretation of the results. Anna Castañé contributed in the setting up of behavioral paradigms. Alberto Prats-Galino contributed with image analysis tools and critically revised the paper. Anna M. Planas helped with the interpretation of the results and critically revised the paper. Tracy D. Farr helped with the general idea of the paper and significantly contributed in the writing process. Guadalupe Soria conceived and designed the study, interpreted the results and wrote the paper.

### Conflict of interest statement

The authors declare that the research was conducted in the absence of any commercial or financial relationships that could be construed as a potential conflict of interest.

## Abbreviations

SHR: spontaneously hypertensive rats
VCI: vascular cognitive impairment
CSV: cerebral small vessel disease
MRI: magnetic resonance imaging
DTI: diffusion tensor imaging
DWI: diffusion weighted imaging
VOI: volume of interest
ADHD: attention deficit and hyperactivity disorder
WKY: Wistar-Kyoto
CC: corpus callosum
mPFC: medial prefrontal cortex
NAcc: nucleus accumbens
OFC: orbitofrontal cortex
adHpc: antero-dorsal hippocampus
FA: fractional anisotropy
AD: axial diffusivity
RD: radial diffusivity
MD: mean diffusivity
CV: cross-validation.
