# Supplementary material for: DWI and complex brain network analysis predicts vascular cognitive impairment in spontaneous hypertensive rats undergoing executive function tests
Source: Front Aging Neurosci. 2014 Jul 23;6:167. doi: 10.3389/fnagi.2014.00167 (PMC4107676; doi:10.3389/fnagi.2014.00167)
Supplement: Supplementary file 1 [file DataSheet1.DOCX]

|  | Fractional Anisotropy | |  |  |  |  |  |  |  |  |  |  |
| --- | --- | --- | --- | --- | --- | --- | --- | --- | --- | --- | --- | --- |
|  | Corpus Callosum | | Medial Prefrontal Cortex | | Hippocampus AD | | Orbitofrontal Cortex | | Striatum | | Nucleus Accumbens | |
|  | *F*-value | *P*-value | *F*-value | *P*-value | *F*-value | *P*-value | *F*-value | *P*-value | *F*-value | *P*-value | *F*-value | *P*-value |
| GxT | F_5,127_= 0.6084 | n.s. | F_5,127_= 0.4641 | n.s. | F_5,127_= 0.2432 | n.s. | F_5,127_= 0.4329 | n.s. | F_5,127_= 1.649 | n.s. | F_5,127_= 0.5911 | n.s. |
| G | F_1,127_= 56.64 | *P*< 0.001 | F_1,127_= 39.27 | *P*< 0.001 | F_1,127_= 4.069 | *P*< 0.05 | F_1,127_= 9.914 | *P*< 0.01 | F_1,127_= 103.1 | *P*< 0.001 | F_1,127_= 51.58 | *P*< 0.001 |
| T | F_5,127_= 2.867 | *P*< 0.05 | F_5,127_= 12.95 | *P*< 0.001 | F_5,127_= 5.794 | *P*< 0.001 | F_5,127_= 1.562 | n.s. | F_5,127_= 12.26 | *P*< 0.001 | F_5,127_= 24.08 | *P*< 0.001 |
|  |  |  |  |  |  |  |  |  |  |  |  |  |
|  | Axial diffusivity | |  |  |  |  |  |  |  |  |  |  |
|  | Corpus Callosum | | Medial Prefrontal Cortex | | Hippocampus AD | | Orbitofrontal Cortex | | Striatum | | Nucleus Accumbens | |
|  | *F*-value | *P*-value | *F*-value | *P*-value | *F*-value | *P*-value | *F*-value | *P*-value | *F*-value | *P*-value | *F*-value | *P*-value |
| GxT | F_5,127_= 2.32 | *P*< 0.05 | F_5,127_= 2.511 | *P*< 0.05 | F_5,127_= 0.616 | n.s. | F_5,127_= 0.8608 | n.s. | F_5,127_= 1.23 | n.s. | F_5,127_= 0.8276 | n.s. |
| G | F_1,127_= 139.2 | *P*< 0.001 | F_1,127_= 97.01 | *P*< 0.001 | F_1,127_= 70.58 | *P*< 0.001 | F_1,127_= 7.049 | *P*< 0.01 | F_1,127_= 12.6 | *P*< 0.001 | F_1,127_= 14.21 | *P*< 0.001 |
| T | F_5,127_= 7.158 | *P*< 0.001 | F_5,127_= 5.584 | *P*< 0.01 | F_5,127_= 4.574 | *P*< 0.001 | F_5,127_= 3.282 | *P*< 0.01 | F_5,127_= 5.159 | *P*< 0.001 | F_5,127_= 2.115 | n.s. |
|  |  |  |  |  |  |  |  |  |  |  |  |  |
|  | Radial diffusivity | |  |  |  |  |  |  |  |  |  |  |
|  | Corpus Callosum | | Medial Prefrontal Cortex | | Hippocampus AD | | Orbitofrontal Cortex | | Striatum | | Nucleus Accumbens | |
|  | *F*-value | *P*-value | *F*-value | *P*-value | *F*-value | *P*-value | *F*-value | *P*-value | *F*-value | *P*-value | *F*-value | *P*-value |
| GxT | F_5,127_= 1.776 | n.s. | F_5,127_= 2.097 | n.s. | F_5,127_= 0.2628 | n.s. | F_5,127_= 1.104 | n.s. | F_5,127_= 1.792 | n.s. | F_5,127_= 1.531 | n.s. |
| G | F_1,127_= 105 | *P*< 0.001 | F_1,127_= 38.02 | *P*< 0.001 | F_1,127_= 101.7 | *P*< 0.001 | F_1,127_= 0.3841 | n.s. | F_1,127_= 0.4501 | n.s. | F_1,127_= 23.86 | *P*< 0.001 |
| T | F_5,127_= 2.174 | n.s. | F_5,127_= 3.134 | *P*< 0.05 | F_5,127_= 2.84 | *P*< 0.05 | F_5,127_= 2.87 | *P*< 0.05 | F_5,127_= 2.414 | *P*< 0.05 | F_5,127_= 2.151 | n.s. |
|  |  |  |  |  |  |  |  |  |  |  |  |  |
|  | Mean diffusivity | |  |  |  |  |  |  |  |  |  |  |
|  | Corpus Callosum | | Medial Prefrontal Cortex | | Hippocampus AD | | Orbitofrontal Cortex | | Striatum | | Nucleus Accumbens | |
|  | *F*-value | *P*-value | *F*-value | *P*-value | *F*-value | *P*-value | *F*-value | *P*-value | *F*-value | *P*-value | *F*-value | *P*-value |
| GxT | F_5,127_= 2.42 | *P*< 0.05 | F_5,127_= 2.664 | *P*< 0.05 | F_5,127_= 0.6024 | n.s. | F_5,127_= 0.7987 | n.s. | F_5,127_= 1.12 | n.s. | F_5,127_= 0.8826 | n.s. |
| G | F_1,127_= 193.2 | *P*< 0.001 | F_1,127_= 76.05 | *P*< 0.001 | F_1,127_= 104.6 | *P*< 0.001 | F_1,127_= 3.203 | n.s. | F_1,127_= 5.458 | *P*< 0.05 | F_1,127_= 29.26 | *P*< 0.001 |
| T | F_5,127_= 4.817 | *P*< 0.001 | F_5,127_= 4.216 | *P*< 0.005 | F_5,127_= 3.079 | *P*< 0.05 | F_5,127_= 2.811 | *P*< 0.05 | F_5,127_= 3.638 | *P*< 0.01 | F_5,127_= 2.356 | *P*< 0.05 |

**Supplementary table 1. Two-way ANOVAs of the regional analysis of DTI scalar maps.** DTI indexes in the selected structures were subjected to repeated measures ANOVA where the between-subjects factor was the rat strain (Wistar or SHR) and time as within-subjects factor of variation (G, group; T, time).

|  | **FA** | **AD** | **RD** | **MD** |
| --- | --- | --- | --- | --- |
| **Nucleus Accumbens** | 0.215 | 0.680 | 0.774 | 0.746 |
| **Caudate Putamen** | 0.739 | 0.611 | 0.482 | 0.503 |
| **Corpus Callosum** | 0.246 | 0.475 | 0.906 | 0.895 |
| **Anterodorsal Hippocampus** | 0.364 | 0.836 | 0.885 | 0.888 |
| **Medial prefrontal Cortex** | 0.746 | 0.718 | 0.510 | 0.597 |
| **Orbitofrontal Cortex** | 0.586 | 0.510 | 0.472 | 0.493 |
|  |  |  |  |  |
|  | **HM** | **GE** | **RE** |  |
| **Whole brain network** | 0.859 | 0.969 | 0.922 |  |

**Supplementary table 2. Area under the receiver operating characteristic’s (ROC) curve of DTI scalar maps and global brain network measurements.** (FA), mean diffusivity (MD), axial diffusivity (AD) and radial diffusivity (RD), (HM) hierarchical modularity, (GE) global efficiency and (RE) regional efficiency.

|  | **WISTAR** | | |  | **SHR** | | |  | |
| --- | --- | --- | --- | --- | --- | --- | --- | --- | --- |
|  | mean |  | SD |  | mean |  | SD |  | |
|  |  |  |  |  |  |  |  |  | |
| **10 weeks** | 100.74 | ± | 20.03 |  | 168.08 | ± | 20.64 | * |  |
|  |  |  |  |  |  |  |  |  |  |
| **26 weeks** | 76.39 | ± | 12.96 |  | 182.88 | ± | 19.96 | * |  |
|  |  |  |  |  |  |  |  |  |  |
| **40 weeks** | 70.22 | ± | 7.32 |  | 187.10 | ± | 24.38 | * |  |

**Supplementary table 3. Ventricle volumes (mm^3^).** Ventricles were manually segmented from T2 relaxometry maps, acquired in the same sessions as DWI. T2 mapping of coronal slices was acquired with a multislice-multi-echo (MSME) sequence by applying 16 different TEs, from 11 to 176 ms, TR = 4764 ms, slice thickness = 1 mm, number of slices = 18, FOV = 40 x 40 mm, and matrix size = 256 x 256 pixels, resulting in a spatial resolution of 0.156 x 0.156 mm in 1.00 mm slice thickness. Asterisk stands for p<0.05 Student’s T test significant difference compared to Wistar rats. (SD: standard deviation).

**Supplementary figure 1**

**
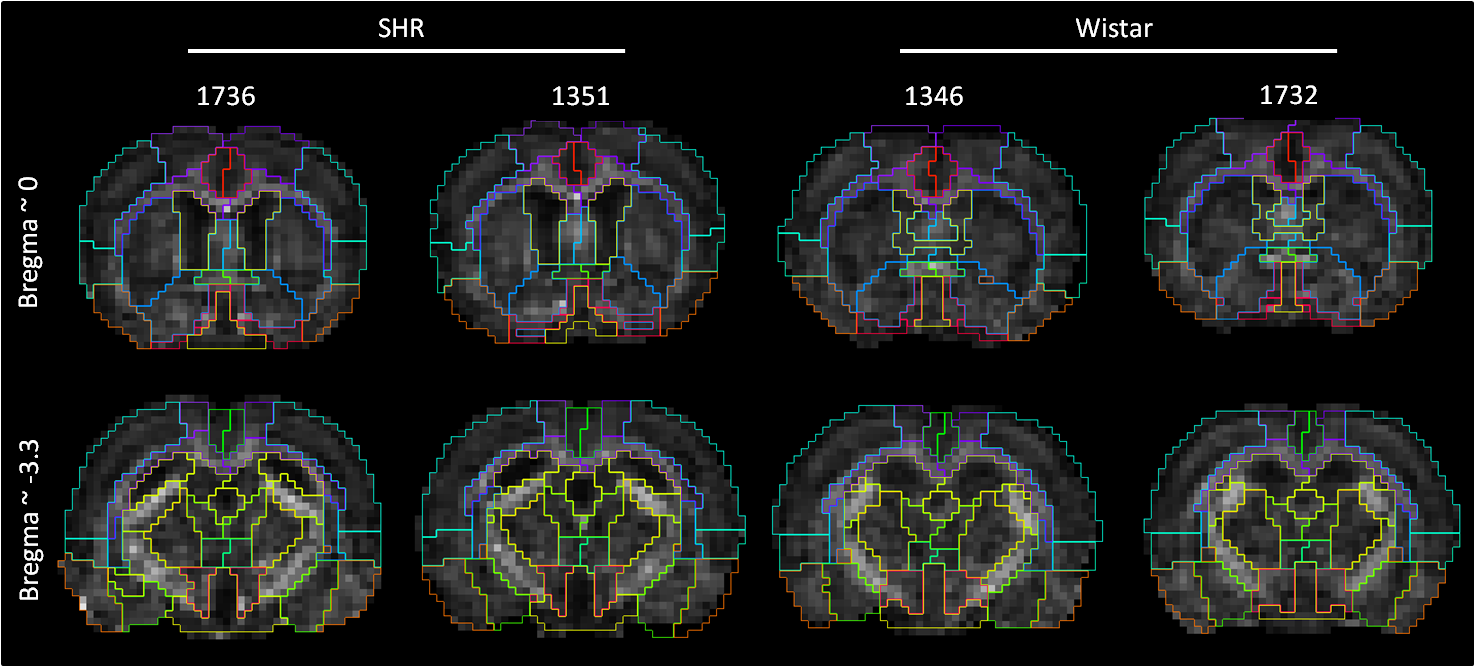
a)**

**
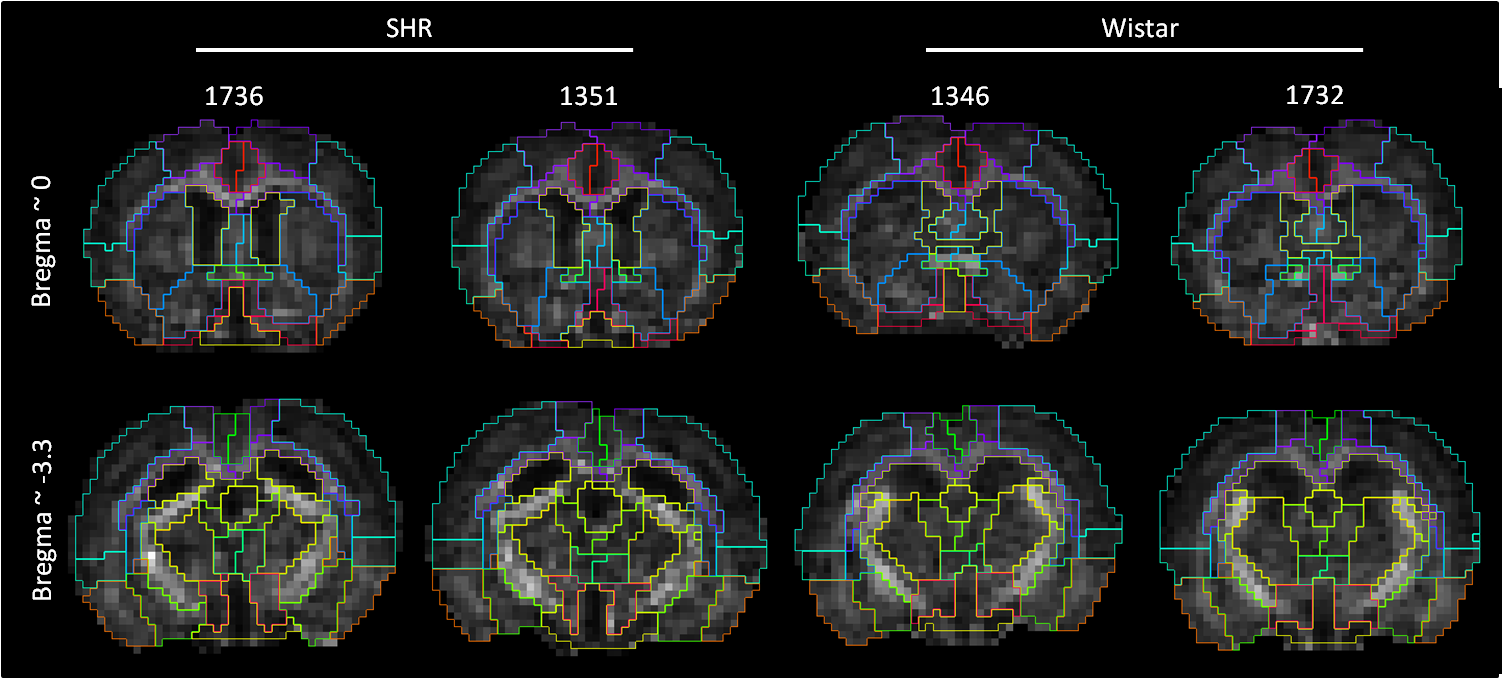
b)**

**
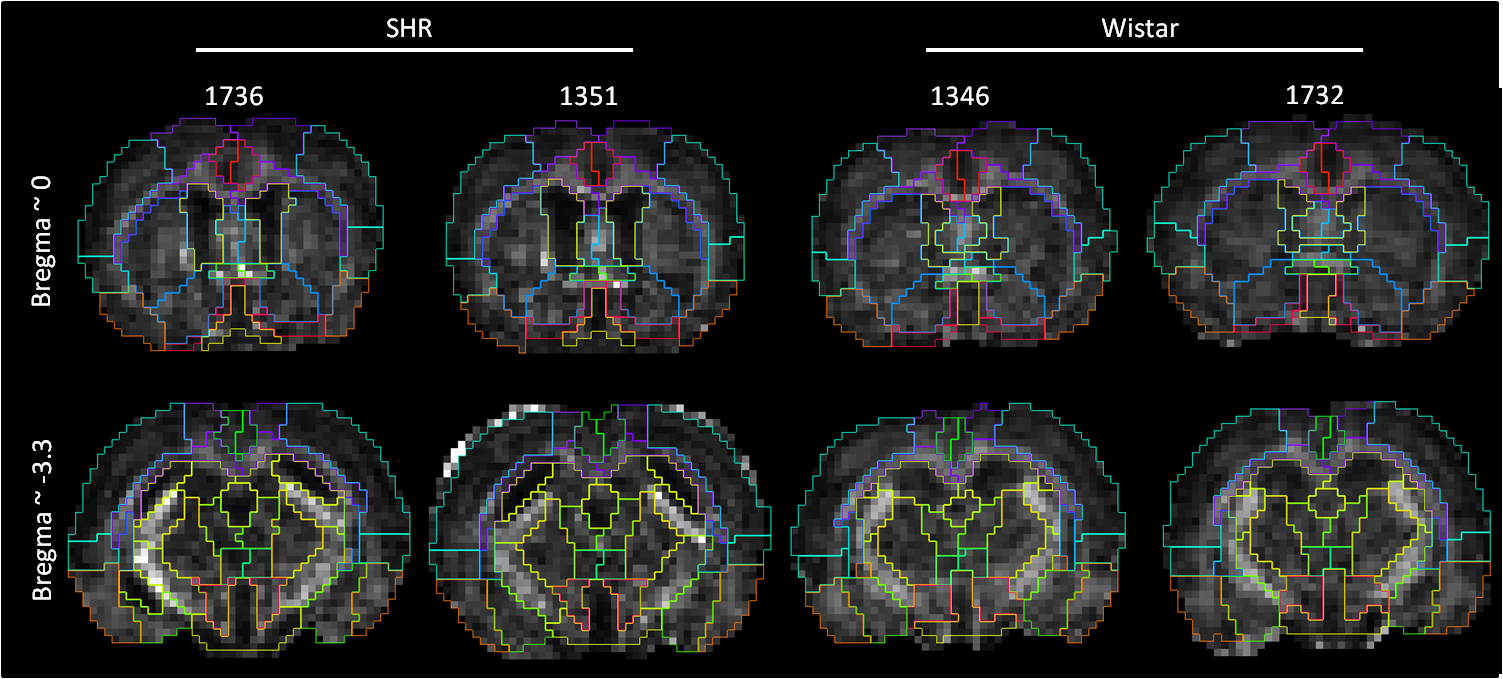
c)**

**Figure S1. Representative examples of rat brain atlas alignment after registration process in 2 Wistar rats and 2 SHRs at the 3 measured time points.**

**Supplementary figure 2**

**
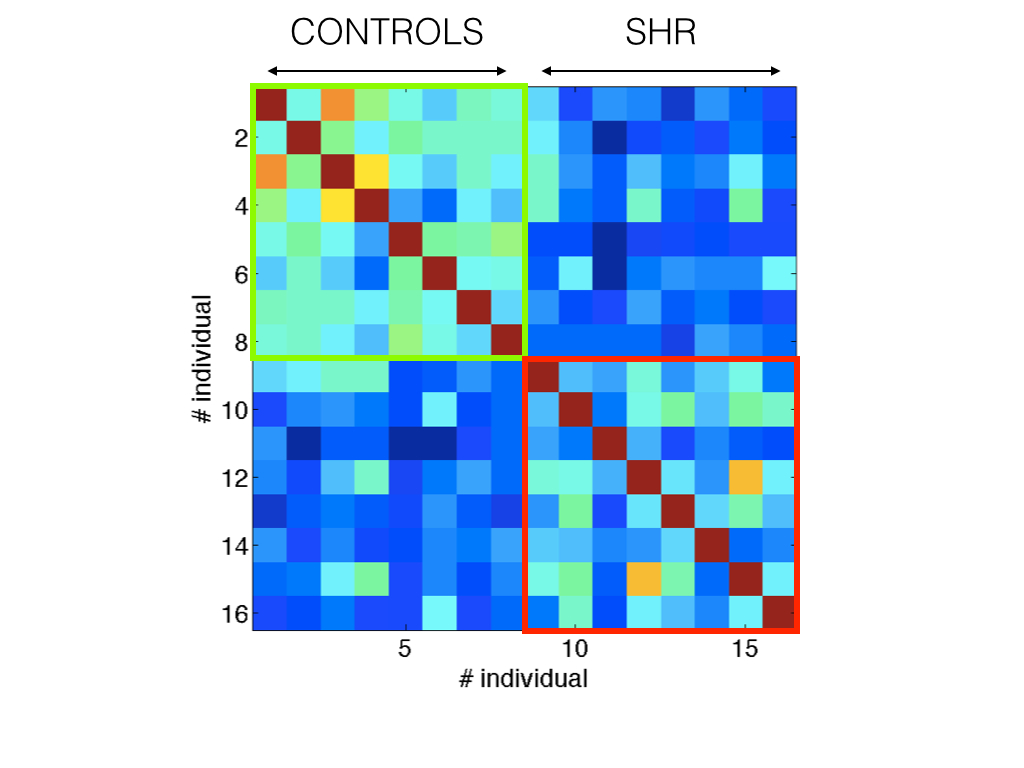
**

**Figure S2. Color-coded matrix representing individual hierarchical modularity at 10 weeks in Wistar rats (controls) and SHRs.** This matrix was constructed in order to provide an overview of the homogeneity between groups**.** The color code ranges from dark blue to dark red, representing from less similarity to highest similarity respectively.

**
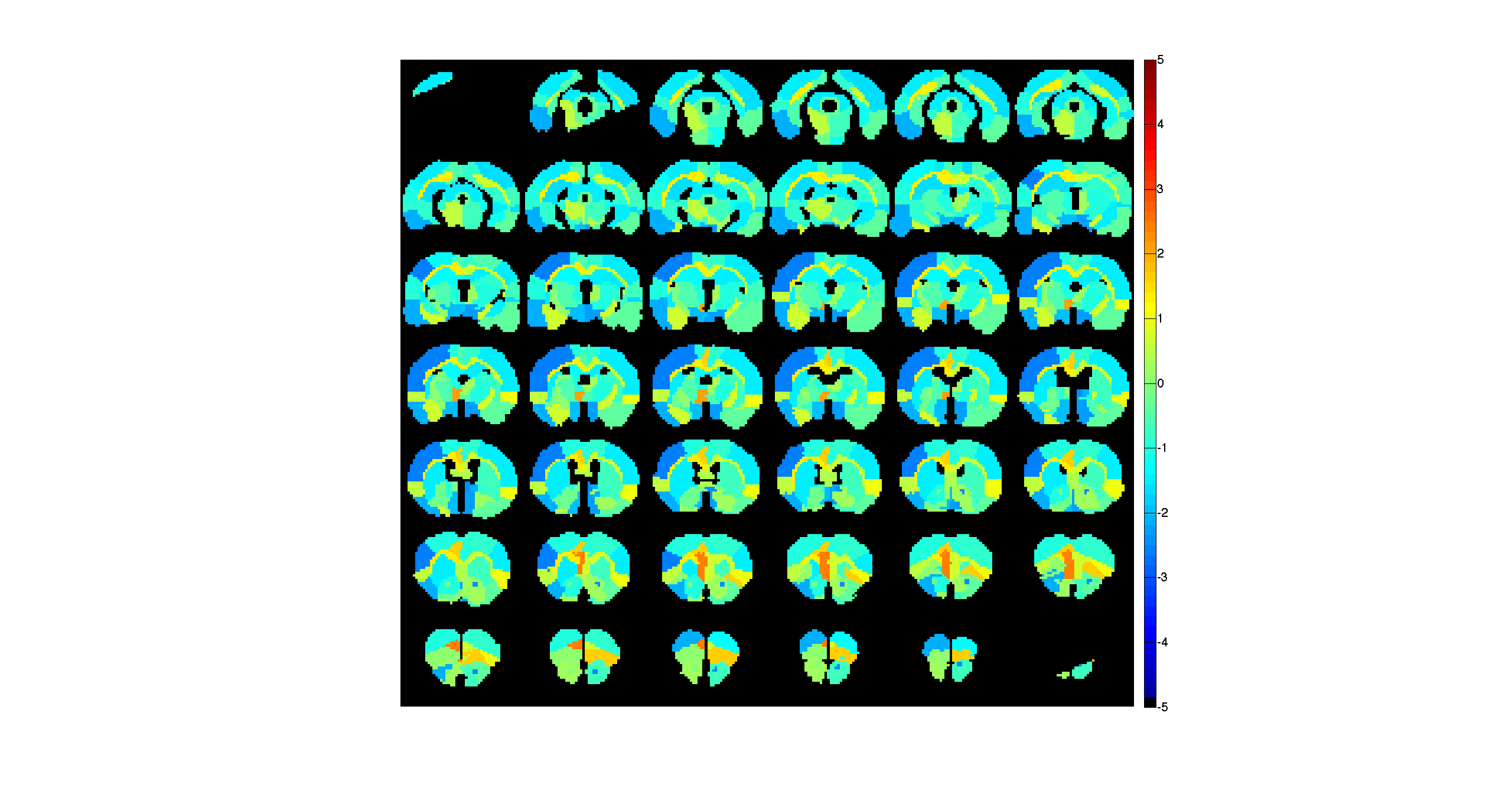

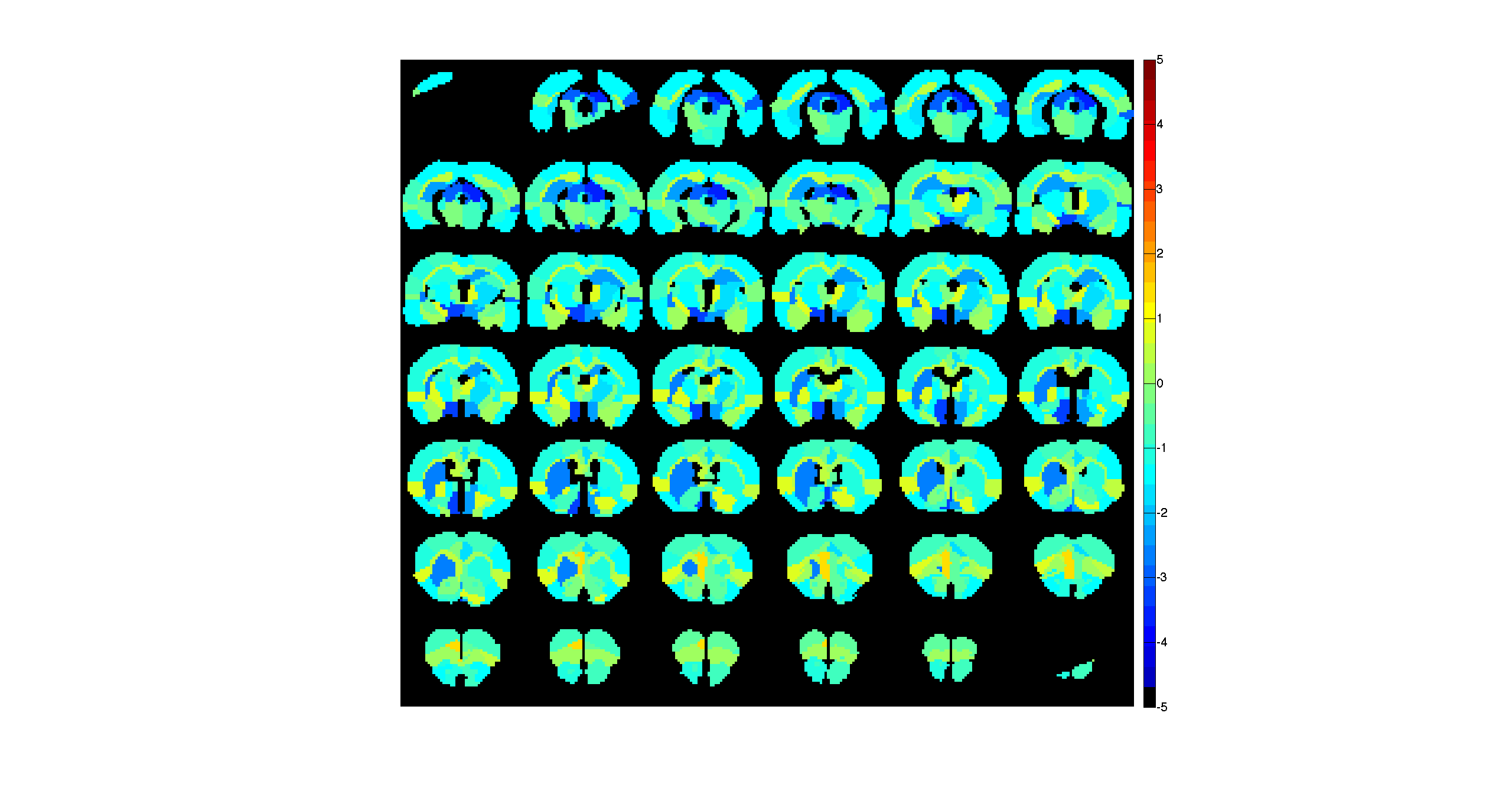
Supplementary Figure 3**

1. **b)**

**
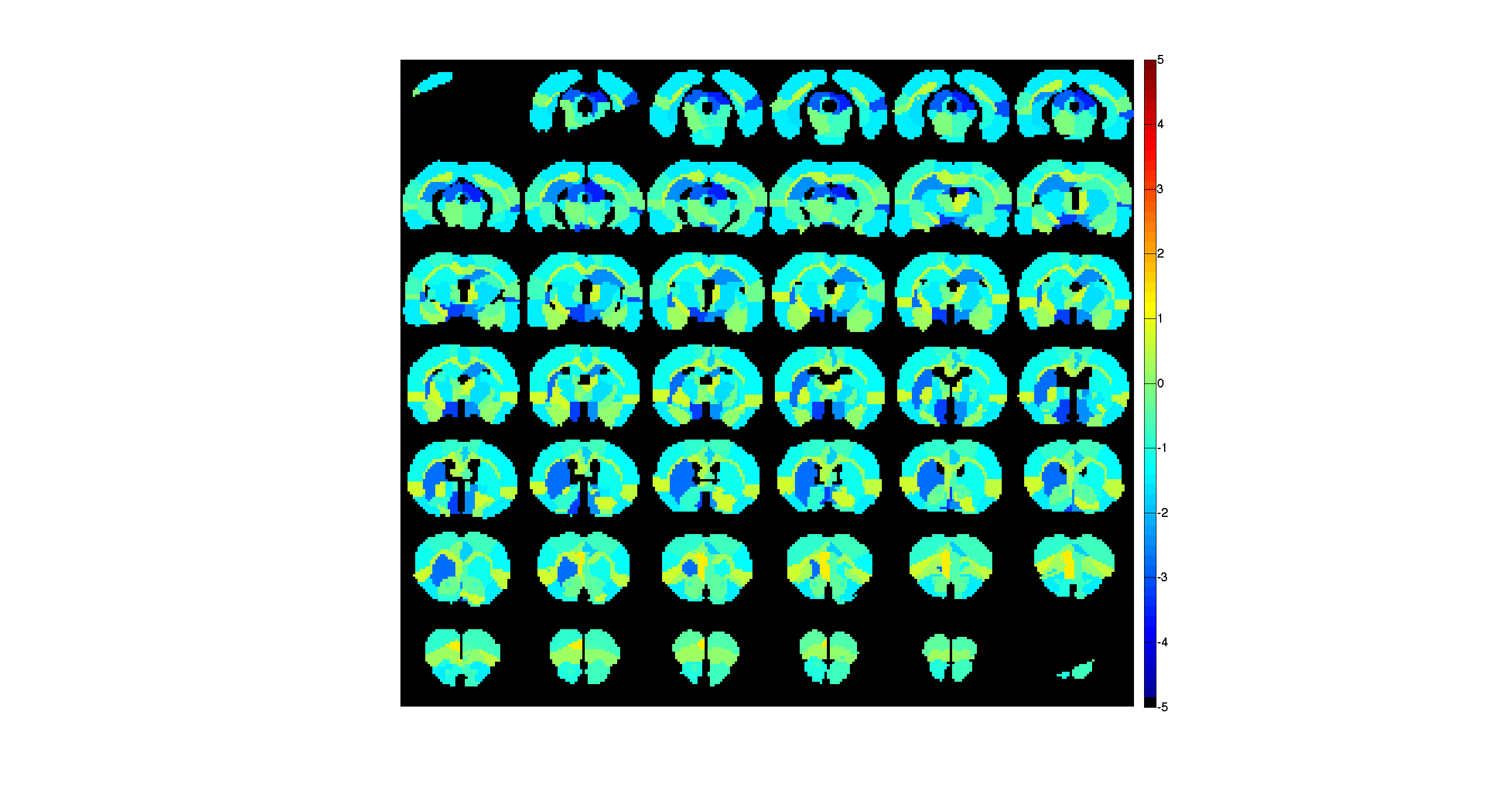
**

**c)**

**Figure S3. Rat brain color coded coronal slices showing the difference of local efficiency for all 84 brain regions at 10 (a), 22 (b) and 40 (c) weeks.** Color coding has been normalized by standard deviation of local efficiency. Significantly altered regions coded in dark blue (significant decrease in SHRs vs Wistar, <-1.96 assuming normal distributions) or orange-red (significant increase in SHRs vs Wistar, >1.96 assuming normal distributions).

**Supplementary Figure 4**

**Figure S4. Representative tractography from Wistar and SHR rats showing tracts emerging from mPFC (A) and tracts between enthorinal cortex and adHpc (B).** It can be observed sparser and less organized connections in the SHR image, probably reflecting the lower hierarchical modularity of those animals and their more random pattern of connections.

**Supplementary Figure 5**

**Figure S5. Receiver operating characteristics (ROC) curves for fractional anisotropy (FA), mean diffusivity (MD), axial diffusivity (AD) and radial diffusivity (RD) DTI scalar maps and the global network measurements (hierarchical modularity, global and regional efficiency).**

**
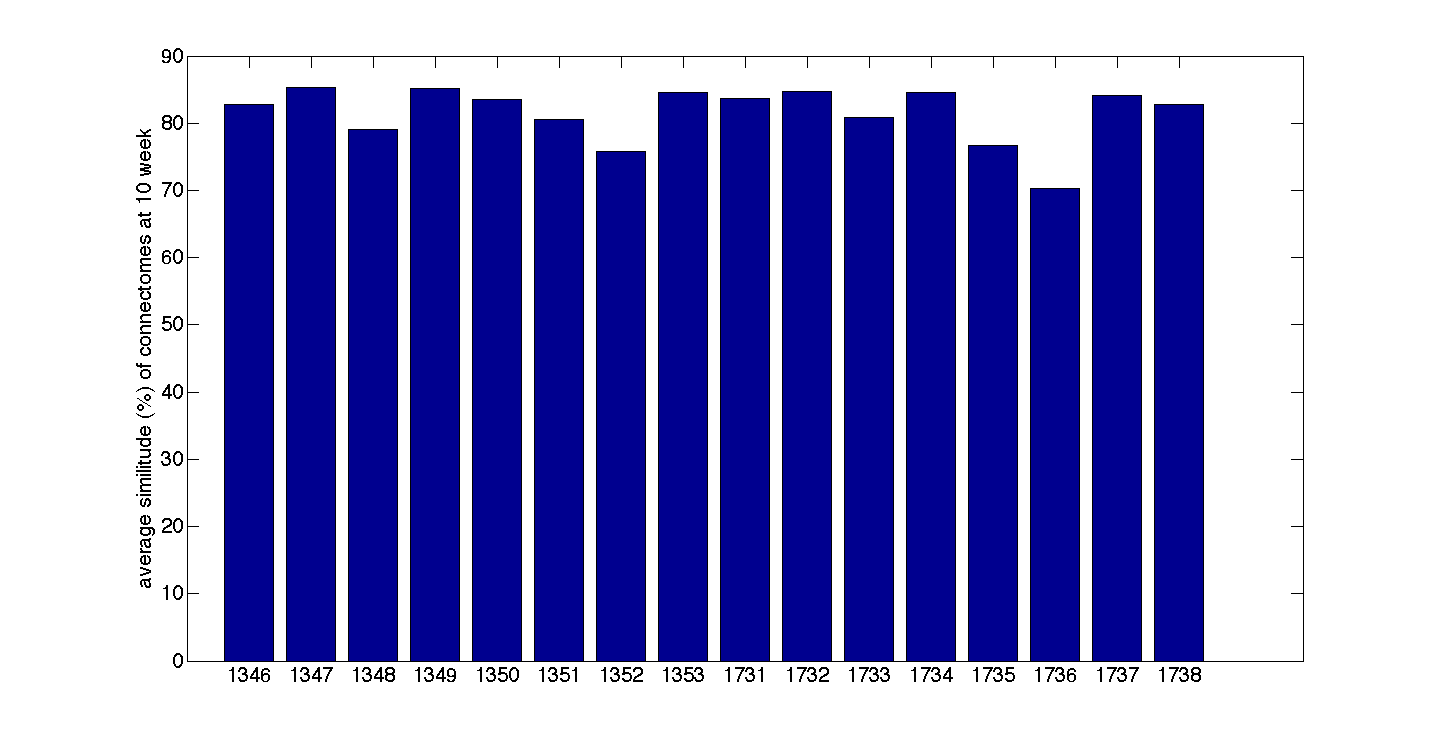
Supplementary Figure 6**

**a)**

**
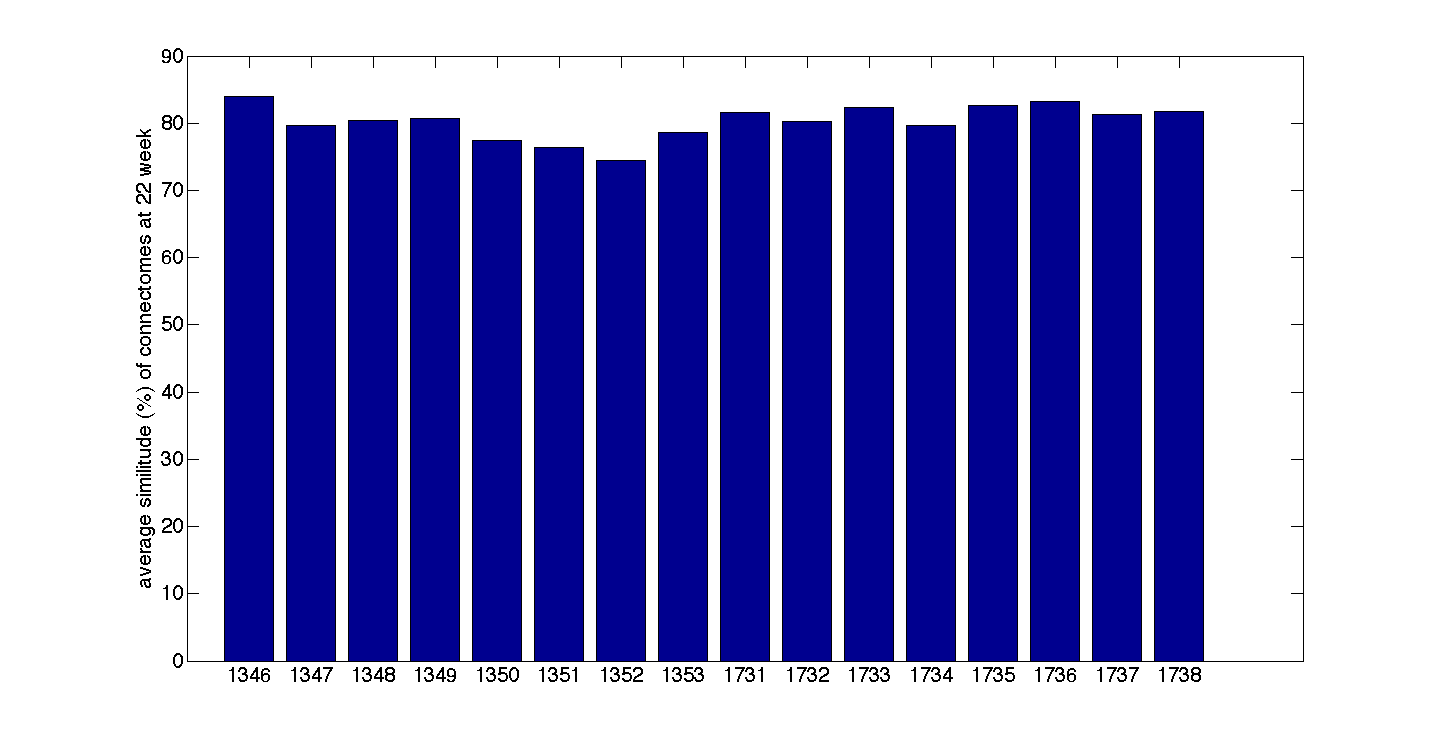
**

**b)**

**
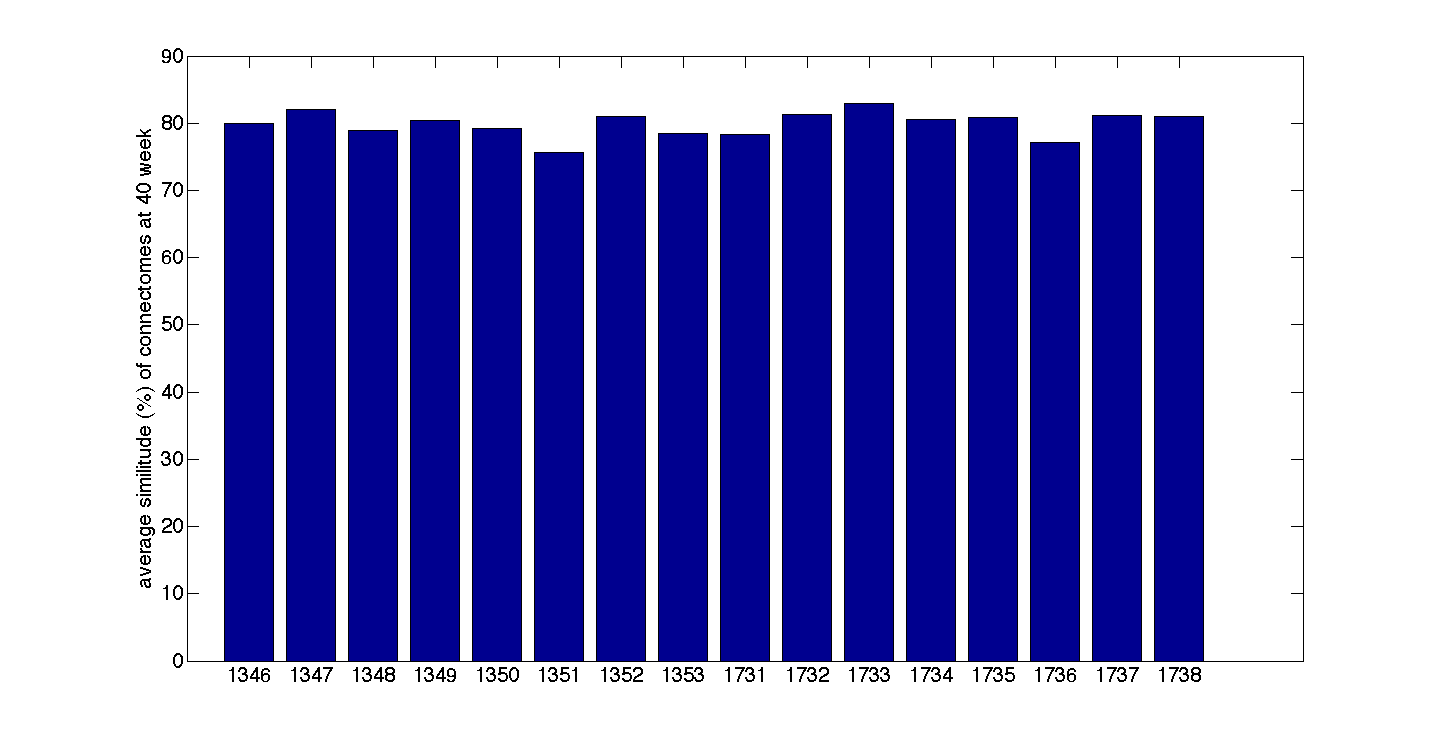
**

**c)**

**Figure S6. Average similitude of connectomes based on normalized cross-correlation.** Variability among subjects is similar between groups and average is around 80%. a) 10 weeks, b) 22 weeks, c) 40 weeks. (Wistar: 1346-1349, 1731-1734; SHR: 1735-1738, 1350-1353).
